# 9-Step synthesis of (−)-larikaempferic acid methyl ester enabled by skeletal rearrangement†

**DOI:** 10.1039/d4cc01462f

**Published:** 2024-05-09

**Authors:** Mario E. Rivera, Lei Li, Aditya Kolisetti, Nina Chi, Mingji Dai

**Affiliations:** a Department of Chemistry, Emory University Atlanta GA 30022 USA mingji.dai@emory.edu 001-404-727-4299; b Department of Pharmacology and Chemical Biology, Emory University Atlanta GA 30022 USA

## Abstract

We report here a concise synthesis of the anti-tumor-promoting (−)-larikaempferic acid methyl ester, a novel and rearranged abietane-type diterpene natural product containing a unique tetracyclic skeleton with a *trans*-hydrindane, an oxabicyclo[3.2.1]octane, and six stereogenic centers. Our synthesis starts with the cheap and abundant abietic acid and features an oxidative C–C bond cleavage followed by a transannular aldol reaction to skeletally rearrange the 6–6–6 tricyclic carbon skeleton of abietic acid to the desired 6–5–7 tricyclic carbon skeleton and an intramolecular oxa-Michael addition to form the oxa bridge. This skeletal rearrangement strategy enabled us to synthesize (−)-larikaempferic acid methyl ester in 9 steps.

## Abstract 2

A 9-step synthesis of (−)-larikaempferic acid methyl ester is achieved *via* a skeletal rearrangement strategy and controlled transannular aldol reaction.

## Graphical abstract

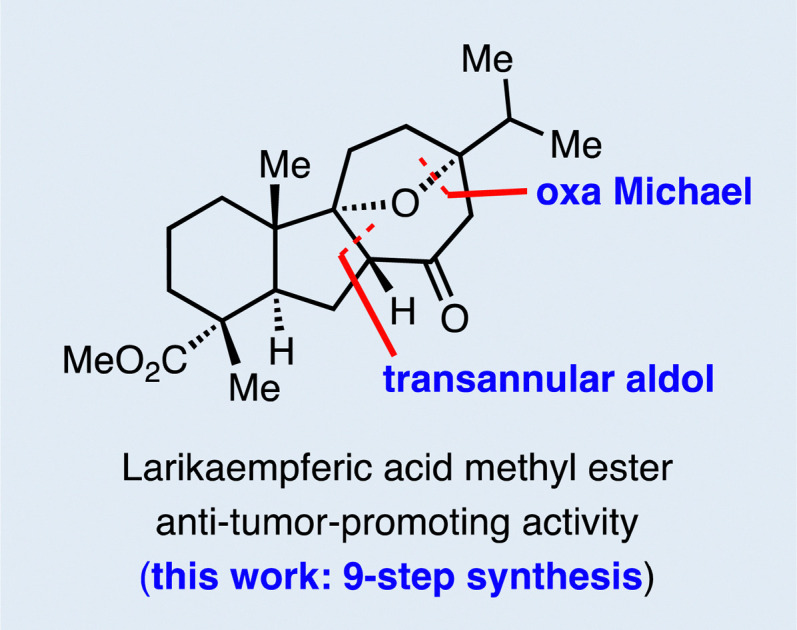

Larikaempferic acid (1, Fig. 1) was first isolated as its methyl ester form (2) by Tanaka and co-workers from the leaves of *Larix kaempferi* in 1999 with 0.0006% isolation yield.^1^ Its structure was elucidated by HRMS, comprehensive NMR analysis, and circular dichroism spectroscopy. It was identified as a structurally rearranged abietane-type diterpene natural product with a unique tetracyclic skeleton featuring a *trans*-hydrindane, an oxabicyclo[3.2.1]octane, and six stereogenic centers. Interestingly, Zhao *et al.* recently discovered larikaempferic acid from the root bark of *Pinus massoniana* as well.^2^ Biologically, larikaempferic acid methyl ester was found to have potent inhibitory effect on Epstein–Barr virus early antigen (EBV-EA) activation in Raji cells induced by 12-*O*-tetradecanoylphorbol 13-acetate (TPA). It's more effective than β-carotene,^3^ which has been heavily investigated in cancer prevention in animal models. Thus, larikaempferic acid methyl ester is a promising anti-tumor-promoting lead for cancer prevention development.

**Fig. 1:**
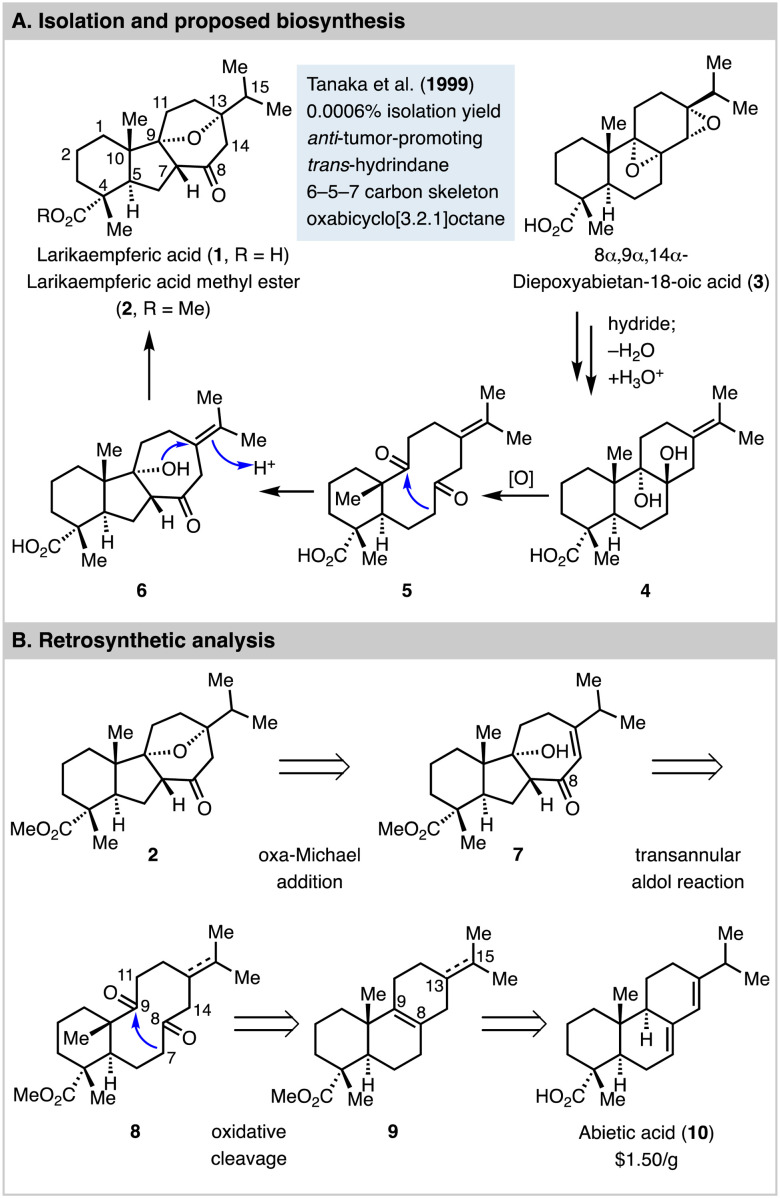
Larikaempferic acid, proposed biosynthesis, and retrosynthetic analysis.

8α,9α,14α-Diepoxyabietan-18-oic acid (3) is another natural product isolated from the same leaves of *Larix kaempferi*, which led Tanaka and Matsunaga to propose 3 as a biosynthesis precursor for larikaempferic acid (1) *via* intermediates including 4, 5, and 6.^4^ As shown in Fig. 1(A), 3 could be converted to 4*via* a sequence of reductive epoxide ring opening with a hydride, dehydration, and a nucleophilic epoxide ring opening with water to form a 1,2-diol (see 4). Oxidative cleavage of the 1,2-diol would convert 4 to 5 with a ten-membered diketone. An enzymatic and stereoselective transannular aldol cyclization would deliver 6 for a subsequent intramolecular etherification to form the oxa bridge and produce larikaempferic acid. While this plausible biosynthesis proposal has not been validated yet, it inspired us to propose a synthetic approach using a transannular aldol reaction^5–11^ as the key step to build the tricyclic carbon skeleton with a *trans*-hydrindane and a *trans* 5,7-fused ring system. As shown in Fig. 1(B), larikaempferic acid methyl ester (2) could be potentially synthesized from enone 7 with the desired 6–5–7 tricyclic ring system *via* an intramolecular oxa-Michael addition. The 6–5–7 tricyclic carbon framework could then be generated *via* a transannular aldol reaction of the *trans* 6,10-fused diketone 8. Diketone 8 could be synthesized from compound 9 with a 6–6–6 tricyclic carbon skeleton, which can be derived from abietic acid (10), a cheap and abundant starting material. At the planning stage, we were aware of a few challenges. First, in addition to the proposed C7–C9 bond formation to form the desired 6–5–7 tricyclic ring system, the transannular aldol reaction could happen between C14 and C9 or C11 and C8 to form two different 6–7–5 tricyclic systems. Second, it might be difficult to control the stereochemistry of the newly formed 5,7-fused ring junction from the transannular aldol reaction, which turned out to be very substrate dependent. Third, it would be ideal to keep the C13–C15 double bond which could migrate into conjugation with the C8 ketone for the oxa-Michael addition, but selective oxidative cleavage of the C8–C9 double bond in presence of the C13–C15 double bond would be challenging. Fourth, if we reduce the C13–C15 double bond, how the stereochemistry at C13 would control the transannular aldol reaction and the following steps was unclear. Overall, we were intrigued by the strategy of structurally rearranging a readily available 6–6–6 tricyclic ring system to the challenging 6–5–7 tricyclic ring system of larikaempferic acid. Herein, we report the details of our explorations which led to a concise 9-step synthesis of (−)-larikaempferic acid methyl ester from abietic acid.

As shown in Scheme 1, our synthesis started from commercially available abietic acid, which can be advanced to 11*via* a reported three-step procedure.^12,13^ As expected, selective oxidative cleavage of the C8–C9 double bond in presence of the C13–C15 double bond turned out to be problematic. Thus, we selectively reduced the C13–C15 double bond with a PtO_2_-catalyzed hydrogenation in presence of the C8–C9 double bond and a 1/1 mixture of inseparable 12a and 12b was obtained in 71% yield. At this stage, oxidative cleavage of the C8–C9 double bond with a combination of RuCl_3_ and NaIO_4_ developed by Sharpless and co-workers^14^ gave a 1/1 mixture of inseparable 13a and 13b in 49% yield. We next used this mixture to explore the transannular aldol reaction. When the mixture was treated with DBU in CH_2_Cl_2_, a 1/1 mixture of 14a and 14b was obtained in 85% total yield and separated. The structures of both 14a and 14b were unambiguously established by X-ray crystallographic analysis (CCDC 2326256 and CCDC 2326255, respectively).† It turned out that a *cis* 5,7-fused ring system with desired stereochemistry at C9 but undesired stereochemistry at C7 was formed during the transannular aldol reaction. Since C7 is potentially epimerizable, we decided to move forward with 14a and 14b for the next Saegusa-Ito oxidation.^15^ Interestingly, while 14b could be converted to enone 15 in 65% yield, 14a failed presumably because the oxoallyl-palladium is formed at the opposite face of the C13 hydrogen atom thus prohibiting the β-hydride elimination to form the enone. Several one-step oxidations including the IBX oxidation^16^ and Mukaiyama dehydrogenation^17^ were explored as well but unsuccessful. After removal of the TMS group on the tertiary alcohol to produce 16, we explored different basic and acidic conditions to epimerize the C7 stereocenter and promote the oxa-Michael addition. Unfortunately, these endeavours were not fruitful. Notably, when 16 was treated with *p*-TsOH, dehydration product 17 was obtained in 70% yield; when it was treated with DBU in toluene at elevated temperature (80 °C), tropone 18 was produced in 42% yield presumably *via* a sequence of dehydration and oxidation with air.

**Scheme 1:**
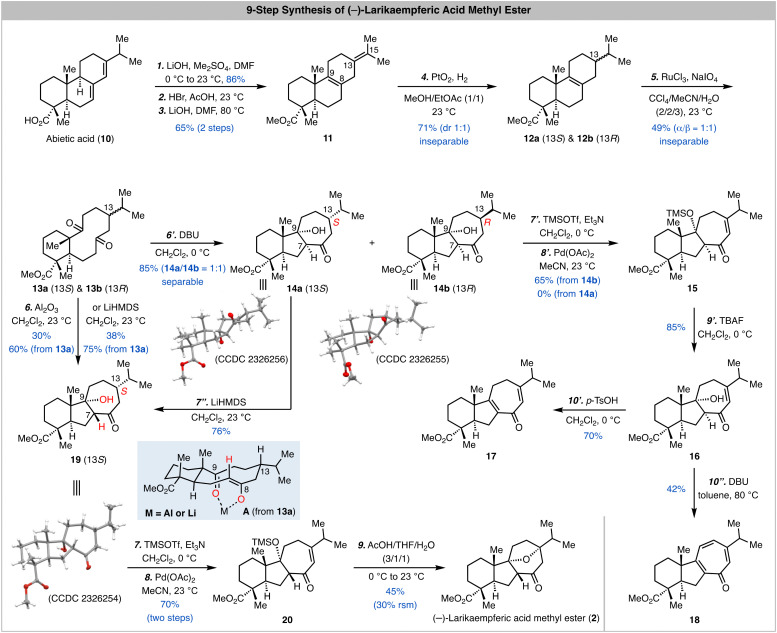
9-Step synthesis of (−)-larikaempferic acid methyl ester and related investigations.

The failure of converting 16 to larikaempferic acid methyl ester (2) indicates that the *trans* ring junction stereochemistry of the 5,7-fused ring system is important for the oxa-Michael addition. Thus, we needed to tune the transannular aldol reaction to provide the desired *trans* 5,7-fused system. We suspected that adding a Lewis acid to chelate with the C8 enolate (*trans* form) and C9 ketone can help to control the transannular aldol reaction (see the chelation model A derived from 13a). To our delight, when the 1/1 mixture of 13a and 13b was treated with Al_2_O_3_,^18,19^ product 19 (CCDC 2326254)† with desired *trans* stereochemistry at the ring junction of the 5,7-fused ring system was obtained in 60% based on the amount of 13a used. Interestingly, 13b didn’t lead to the formation of the corresponding transannular aldol product but decomposed, which is presumably due to the high energy of the transition state with the isopropyl resides in the pseudo axial position (structure not shown; see model A but switch the position of the hydrogen atom and isopropyl group on C13). We also discovered that treating the mixture of 13a and 13b with LiHMDS gave similar result and 19 was obtained in higher yield (75% based on 13a). During this reaction, we observed that both 19 and 14a were produced at an early stage and 19 was the dominant product at the end of the reaction, which indicates that 14a could be converted to 19*via* a sequence of retro–aldol reaction and chelation controlled transannular aldol cyclization. Indeed, when 14a was subjected to the LiHMDS conditions, 19 was produced in 76% yield. Notably, 14b didn’t undergo a similar process to epimerize the C7 stereocenter, but elimination of the C9 alcohol.

With a better understanding of the transannular aldol reaction and 19 in hand, we moved forward to complete the synthesis of larikaempferic acid methyl ester (2). Saegusa-Ito oxidation of 19 occurred smoothly to deliver enone 20 in 70% yield over two steps. We then developed a one-step procedure to remove the TMS group and trigger the oxa-Michael addition. When 20 was treated with a mixture of AcOH/THF/H_2_O (3/1/1), (−)-larikaempferic acid methyl ester was obtained in 45% yield. The NMR, Mass Spec, and optical rotation data of our synthetic sample matched well with the ones reported for the natural sample.

In summary, using a skeletal rearrangement^20^ and transannular cyclization strategy,^21,22^ we developed an efficient synthesis of (−)-larikaempferic acid methyl ester from abietic acid. The key steps include an oxidative C8–C9 double bond cleavage to deliver a 10-membered diketone, a chelation-controlled transannular aldol cyclization to build the *trans* 5,7-fused ring system, and an oxa-Michael addition to form the oxabicyclo[3.2.1]octane. These enabling transformations led us to (−)-larikaempferic acid methyl ester in 9 steps. In addition, this synthesis provides support to the proposed biosynthetic pathway and suggests the oxa-Michael reaction could be an alternative process for the oxa bridge formation.

This work was financially supported by NIH GM128570. We thank Dr John Bacsa for collecting the XRD data for 14a (CCDC 2326256), 14b (CCDC 2326255), and 19 (CCDC 2326254).† We also thank Dr Bing Wang and Dr Shaoxiong Wu for NMR measurements and Dr Frederick Strobel for high resolution Mass Spectrometry analysis.

## Conflicts of interest

There are no conflicts to declare.

## Supplementary Material

CC-060-D4CC01462F-s001

CC-060-D4CC01462F-s002
