# Supplementary material for: 9-Step synthesis of (−)-larikaempferic acid methyl ester enabled by skeletal rearrangement
Source: Chem Commun (Camb). 2024 May 9;60(56):7164–7. doi: 10.1039/d4cc01462f (PMC11232478; doi:10.1039/d4cc01462f)
Supplement: CC-060-D4CC01462F-s001 [file CC-060-D4CC01462F-s001.pdf]

Electronic Supplementary Material (ESI) for ChemComm.
This journal is © The Royal Society of Chemistry 2024

Supporting Information

9-Step Synthesis of (−)-Larikaempferic Acid Methyl Ester Enabled by Skeletal Rearrangement

Mario E. Rivera, Lei Li, Aditya Kolisetti, Nina Chi, and Mingji Dai*

Department of Chemistry, Emory University, Atlanta, GA 30322, United States
Department of Pharmacology and Chemical Biology, Emory University, Atlanta, GA 30322, United States

Table of Contents

Part 1. Experimental procedures and spectra data..S3

Part 2. X-Ray structure and analysis data..S15

Part 3. References..S17

Part 4. ^{1}H and ^{13}C NMR spectra...S18

Part 1. Experimental Procedures and Experimental Data

General Methods: Reactions were performed using standard syringe techniques under argon atmosphere unless stated otherwise. Starting materials and reagents were used as received from suppliers. Dichloromethane (CH_2Cl_2), acetonitrile (MeCN), tetrahydrofuran (THF), dimethylformamide (DMF), and toluene were purified by passing the previously degassed solvents through activated alumina columns. Flash chromatography was performed using silica gel (230-400 mesh). Thin layer chromatography (TLC) was performed using glass-backed silica plates (Silicycle). NMR spectra were recorded on a Bruker AV-400, 500 or 600 spectrometers at room temperature. Chemical shifts (in ppm) are given in reference to the solvent signal [^{1}H NMR: $CDCl_3$ (7.26); ^{13}C NMR: $CDCl_3$ (77.16)]. ^{1}H NMR data are reported as follows: chemical shift (δ ppm), multiplicity (s = singlet, d = doublet, t = triplet, q = quartet, quin = quintuplet, m = multiplet, br = broad), coupling constant (Hz), and integration. ^{13}C NMR data are reported in terms of chemical shift and multiplicity. IR data were recorded on a Thermo Nicolet Nexus 470 FTIR. Room temperature is around 23 °C.

Experimental Procedures

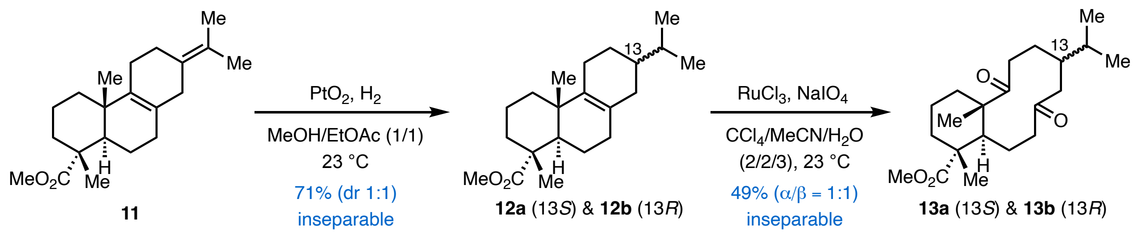

To a flame-dried 50 mL round bottom flask under an argon balloon was added diene **11**[1] (200 mg, 0.632 mmol, 1.0 equiv.) followed by a mixture (1:1) of anhydrous methanol (10 mL) and ethyl acetate (10 mL) at room temperature. PtO_2 (3.8 mg, 0.0126 mmol) was then added. The mixture was stirred for 5 min before the argon balloon was removed. The reaction mixture was bubbled with H_2 gas for 5 min then stirred under H_2 atmosphere overnight. After 16 h, the reaction mixture was filtered through a pad of celite and washed with ethyl acetate (3 x 15 mL). The resulting solution was concentrated in vacuo to yield a clear yellow oil which was purified via flash column chromatography (hexanes/ethyl acetate, 99:1 to 90:10) to obtain an inseparable mixture of isomers **12a**/**12b** together with over reduced byproducts (71% yield of **12a**/**12b** based on NMR analysis of this mixture). This mixture was not further purified and used directly in the next step.

To a flame-dried 250 mL round bottom flask, $RuCl_3$ (19.7 mg, 0.095 mmol, 5 mol %) was added and stirred in water (66 mL). In four 20-min intervals, $NaIO_4$ was added (1.0 g each, 2.5 equiv.). After the third interval, MeCN was added (45 mL) and subsequently the mixture of **12a/12b** (600 mg, 1.88 mmol), dissolved in CCl_4 (45 mL) was added dropwise. Once the $NaIO_4$ was added for the fourth time, the flask was fitted with a glass-stopper and left stirring at room temperature overnight. After 16 h, another 5.0 equiv. of $NaIO_4$ (2.01 g) was added in 2-hour intervals as 2.5 equiv. each interval. The reaction was then quenched with ethanol (11 mL), passed through a celite plug and extracted with DCM (70 mL). The organic layers were combined and washed once with sodium thiosulfate (150 mL), dried over anhydrous Na_2SO_4, filtered, concentrated, and purified via flash column chromatography (hexanes/ethyl acetate, 4:1 to 2:1) to give an inseparable mixture of **13a** and **13b** as a white amorphous solid (322 mg, 49%).

13a and **13b** were characterized as a 1:1 mixture with different conformers.

^{1}H NMR (400 MHz, $CDCl_3$) δ 3.79 (s, 3H), 3.78 (s, 3H), 2.88 (d, J = 8.0 Hz, 1H), 2.51-2.49 (m, 2H), 2.52-2.46 (m, 2H), 2.37-1.18 (m, 42H), 0.92-0.78 (m, 15H);

^{13}C NMR (101 MHz, CDCl$_3$) δ 217.1, 213.0, 178.9, 178.4, 53.5, 52.7, 52.3, 51.5, 48.1, 47.1, 46.7, 38.9, 36.9, 36.6, 35.9, 34.7, 33.5, 33.2, 29.8, 25.5, 24.8, 22.8, 22.4, 19.8, 19.7, 19.1, 19.0, 17.6, 17.4, 17.3, 16.5;

HRMS (APCI): *m/z* calc for $C_{21}H_{35}O_4$ $^+$[M+H]$^+$: 351.2530, found 351.2522;

IR (film): 2952, 2872, 1725, 1703, 1468, 1432, 1388, 1368, 1243, 1213, 1165, 1081, 1032 cm^{-1}.

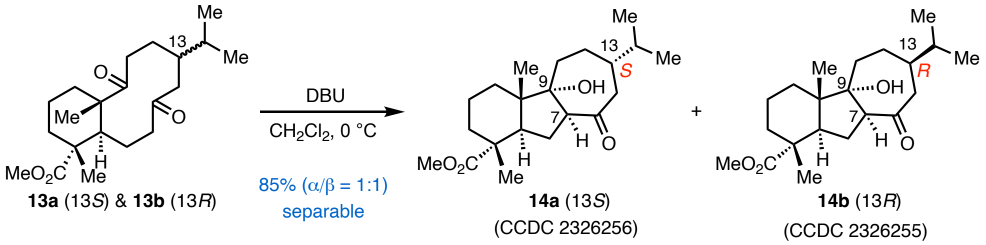

To a flamed-dried vial was added a 1:1 mixture of diketone **13a**/**13b** (255 mg, 0.728 mmol.) in CH_2Cl_2 (8.0 mL) via syringe under argon atmosphere. The resulting solution was stirred at 0 °C for 15 min. DBU (0.135 mL, 0.904 mmol.) was added dropwise. The reaction was stirred at 0 °C. After 90 min, the reaction mixture was warmed to room temperature and quenched with saturated NH_4Cl (3 mL, dropwise) and extracted with CH_2Cl_2 (3 x 10 mL). The combined organic layers were dried over anhydrous Na_2SO_4, filtered, and concentrated in vacuo to obtain a white foamy solid which was purified via flash column chromatography (pentane/ethyl acetate, 4:1 to 2:1). Two separable isomers **14a** and **14b** (**14a**:**14b** = 1:1, total 216 mg, 85%) were obtained as white solids.

Compound 14a:

^{1}H NMR (400 MHz, CDCl$_3$) *δ* 3.64 (s, 3H), 3.06 (t, *J* = 8.6 Hz, 1H), 2.45 (d, *J* = 9.3 Hz, 2H), 2.25 (dd, *J* = 13.9, 5.7 Hz, 1H), 2.08 (ddd, *J* = 13.9, 12.2, 8.6 Hz, 1H), 1.90 – 1.81 (m, 1H), 1.76 – 1.56 (m, 8H), 1.52 (m, 1H), 1.46 – 1.36 (m, 3H), 1.28 (s, 3H), 0.90 (d, *J* = 6.9 Hz, 3H), 0.88 (d, *J* = 6.9 Hz, 3H), 0.77 (s, 3H);

^{13}C NMR (100 MHz, CDCl$_3$) δ 212.4, 178.9, 83.6, 61.6, 52.0, 49.5, 48.6, 45.7, 45.6, 45.2, 35.7, 33.9, 33.5, 31.5, 25.6, 25.0, 19.8, 19.3, 18.7, 17.5, 16.4;

HRMS (APCI): *m/z* calc for $C_{21}H_{35}O_4$ $^+$[M+H]$^+$: 351.2529, found 351.2522;

IR (film): 3472, 2951, 2923, 1725, 1701, 1455, 1385, 1235, 1187, 1136, 1088, 1052, 1007, 976, 943 cm^{-1}.

Compound 14b:

^{1}H NMR (400 MHz, $CDCl_3$) δ 3.64 (s, 3H), 3.16 (dd, J = 8.8, 7.6 Hz, 1H), 2.42 (m, 1H), 2.29 (dd, J = 13.9, 5.6 Hz, 1H), 2.23 – 2.13 (m, 2H), 1.86 (m, 1H), 1.72 (m, 1H), 1.66 – 1.40 (m, 11H), 1.27 (s, 3H), 0.92 (d, J = 6.7 Hz, 3H), 0.89 (d, J = 6.7 Hz, 3H), 0.68 (s, 3H);

^{13}C NMR (101 MHz, $CDCl_3$) δ 211.9, 178.9, 84.0, 59.7, 52.0, 49.0, 45.5, 45.1, 44.6, 37.0, 35.9, 32.3, 30.9, 28.9, 25.6, 23.3, 19.9, 19.7, 18.6, 17.3, 15.9;

HRMS (ESI): *m/z* calc for $C_{21}H_{34}O_4Na^+$ $[M+Na]^+$: 373.2349, found 373.2346;

IR (film): 3492, 2951, 2923, 2868, 1725, 1698, 1456, 1386, 1368, 1329, 1243, 1164, 1140, 1116, 1051, 996, 940, 908 cm^{-1}.

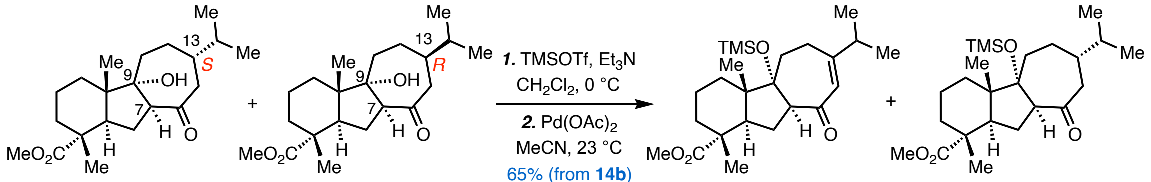

14a (13*S*) **14b** (13*R*) **15** **15a**

To a flame-dried vial under argon atmosphere, was added a 1:1 mixture of **14a** and **14b** (116 mg, 0.33 mmol,) and CH_2Cl_2 (7 mL). The mixture was stirred at 0 °C for 10 min. Anhydrous triethylamine (0.369 mL, 2.65 mmol, 8.0 equiv.) was added followed by dropwise addition of TMSOTf (0.240 mL, 1.32 mmol, 4.0 equiv.). This mixture was stirred at 0 °C for 1 h before it was warmed to room temperature, and quenched with saturated $NaHCO_3$ (4 mL), extracted with CH_2Cl_2 (3 x 5 mL). The organic layers were dried with anhydrous Na_2SO_4, filtered, and concentrated in vacuo to give a clear oil which was used in the next step without further purification.

The above crude silyl enol ether intermediate was flushed with argon and dissolved in MeCN (9 mL). $Pd(OAc)_2$ (133 mg, 0.592 mmol.) was quickly added. The reaction mixture was stirred overnight at room temperature before it was filtered through celite, washed with ethyl acetate,

concentrated, and purified via flash column chromatography (hexanes/ethyl acetate, 3:1) to yield a yellow oil that crystallized to a yellow-white solid **15** (45 mg, 65% from **14b**) and protected alcohol **15a** (56 mg, 71% from **14a**).

Compound 15:

^{1}H NMR (400 MHz, CDCl$_3$) δ 5.94 (d, J = 2.6 Hz, 1H), 3.64 (s, 3H), 2.99 (m, 1H), 2.67 (ddt, J = 19.0, 11.0, 1.9 Hz, 1H), 2.40 – 2.32 (m, 3H), 2.27 (m, 1H), 1.76 (ddd, J = 13.9, 6.3, 1.9 Hz, 1H), 1.69 – 1.52 (m, 7H), 1.43 (m, 1H), 1.28 (s, 3H), 1.09 (d, J = 6.8 Hz, 3H), 1.07 (d, J = 6.8 Hz, 3H), 0.66 (s, 3H), 0.19 (s, 9H);

^{13}C NMR (101 MHz, CDCl$_3$) δ 201.7, 178.9, 165.5, 126.6, 89.7, 62.4, 51.8, 49.5, 45.6, 45.0, 37.38, 36.0, 33.3, 31.5, 28.3, 23.1, 21.7, 21.2, 18.8, 17.0, 16.6, 2.70;

HRMS (APCI): *m/z* calc for $C_{24}H_{41}O_4Si^+$ $[M+H]^+$: 421.2768, found 421.2773;

IR (film): 2950, 2922, 1727, 1664, 1457, 1386, 1322, 1248, 1219, 1183, 1162, 1087, 1057, 1020, 996, 856, 838, 753, 408 cm^{-1}.

Compound 15a:

^{1}H NMR (400 MHz, CDCl$_3$) δ 3.64 (s, 3H), 3.14 (t, J = 8.4 Hz, 1H), 2.47 – 2.37 (m, 2H), 2.27 (dd, J = 14.0, 6.0 Hz, 1H), 2.08 (ddd, J = 14.0, 12.4, 8.2 Hz, 1H), 1.80 – 1.56 (m, 8H), 1.56 – 1.36 (m, 3H), 1.31 (m, 1H), 1.27 (s, 3H), 1.24 (m, 1H), 0.90 (d, J = 6.8 Hz, 3H), 0.88 (d, J = 6.8 Hz, 3H), 0.74 (s, 3H), 0.23 (s, 9H);

^{13}C NMR (101 MHz, CDCl$_3$) δ 212.2, 179.0, 88.8, 62.3, 51.8, 49.7, 49.6, 45.6, 45.4, 45.2, 36.0, 33.6, 33.5, 32.2, 29.8, 26.2, 24.8, 19.7, 19.2, 18.8, 17.3, 16.6, 3.0;

HRMS (ESI): *m/z* calc for $C_{24}H_{42}O_3NaSi^+$ $[M+Na]^+$: 445.2744, found 445.2742;

IR (film): 2952, 2917, 2849, 1727, 1704, 1461, 1386, 1250, 1146, 1118, 1080, 1023, 866, 838, 738 cm^{-1}.

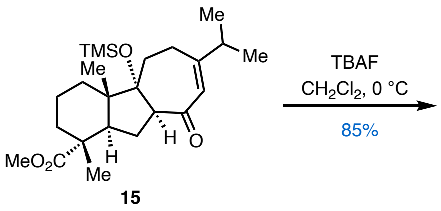

To a flame dried vial containing enone **15** (326 mg, 0.78 mmol) under argon atmosphere was added CH_2Cl_2 (3 mL). Tetrabutylammonium fluoride (1 M in THF, 0.85 µL, 1.1 equiv.) was added dropwise at 0 °C. The resulting reaction mixture was stirred at 0 °C until TLC showed complete consumption of the starting material (about 1h). The reaction mixture was filtered through a silica plug and rinsed with ethyl acetate (9 mL). The organic solution was dried over anhydrous sodium sulfate, filtered, and concentrated under reduced pressure. The residue was purified via flash column chromatography (hexane/ethyl acetate, 5:1 to 1:1) to obtain alcohol **16** as a white solid (230 mg, 0.660 mmol, 85%).

^{1}H NMR (400 MHz, $CDCl_3$) *δ* 5.96 (d, J = 3.0 Hz, 1H), 3.65 (s, 3H), 2.93 – 2.84 (m, 2H), 2.45 – 2.35 (m, 3H), 2.29 (m, 1H), 1.83 – 1.57 (m, 6H), 1.54 (m, 1H), 1.47 – 1.40 (m, 2H), 1.32 (m, 1H), 1.30 (s, 3H), 1.09 (d, J = 6.8 Hz, 3H), 1.08 (d, J = 6.8 Hz, 3H), 0.71 (s, 3H);

^{13}C NMR (101 MHz, $CDCl_3$) δ 201.6, 178.9, 166.4, 126.5, 84.6, 61.8, 52.0, 48.6, 45.6, 45.3, 37.5, 35.8, 33.4, 31.1, 27.6, 23.0, 21.6, 21.2, 18.7, 17.2, 16.3;

HRMS (APCI): *m/z* calc for $C_{21}H_{33}O_4^+$ $[M+H]^+$: 349.2373, found 349.2375;

IR (film): 3479, 2920, 2869, 1725, 1644, 1456, 1432, 1385, 1327, 1296, 1236, 1220, 1181, 1113, 1070, 1051, 1031, 980, 920, 890, 856 cm^{-1}.

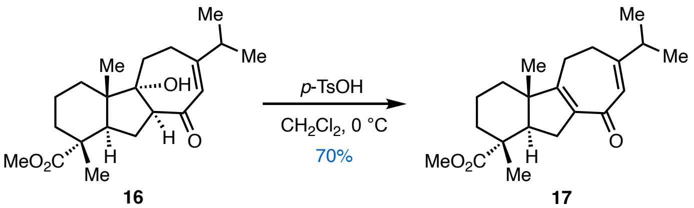

To a flame dried vial containing alcohol **16** (17 mg, 0.05 mmol, 1.0 equiv.) under argon atmosphere was added CH_2Cl_2 (0.5 mL). The mixture was stirred at 0 °C for 5 min before *p*-TsOH (8.4 mg, 0.05 mmol, 1.0 equiv.) in CH_2Cl_2 (0.5 mL) was added dropwise. The reaction mixture was first stirred at 0 °C for 30 min, then warmed up to room temperature for 1 h until complete consumption of starting material. Reaction mixture was quenched with aqueous sodium bicarbonate (0.5 mL) and extracted with dichloromethane (3 x 2 mL). The combined organic layers were washed with brine, dried over anhydrous Na_2SO_4, filtered, and concentrated. The residue was purified via flash column chromatography (hexane/ethyl acetate, 3:1) to afford **17** a yellowish foamy solid (11.3 mg, 70%).

^{1}H NMR (400 MHz, $CDCl_3$) δ 5.99 (d, J = 0.9 Hz, 1H), 3.66 (s, 3H), 2.46 – 2.38 (m, 2H), 2.38 – 2.29 (m, 4H), 2.04 (dd, J = 11.8, 6.5 Hz, 1H), 1.84 – 1.63 (m, 5H), 1.40 (m, 1H), 1.29 (s, 3H), 1.09 – 1.07 (m, 6H), 0.96 (s, 3H);

^{13}C NMR (101 MHz, $CDCl_3$) δ 190.9, 178.2, 166.5, 162.1, 138.4, 127.7, 52.1, 51.8, 50.4, 45.4, 38.5, 35.9, 34.3, 30.4, 29.6, 24.4, 21.1, 20.9, 19.0, 17.8, 16.9;

HRMS (APCI): *m/z* calc for $C_{21}H_{31}O_3^+$ $[M+H]^+$: 331.2267, found 331.2267;

IR (film): 2918, 2849, 1726, 1640, 1600, 1453, 1377, 1249, 1172, 1121, 1057, 887, 886 cm^{-1}.

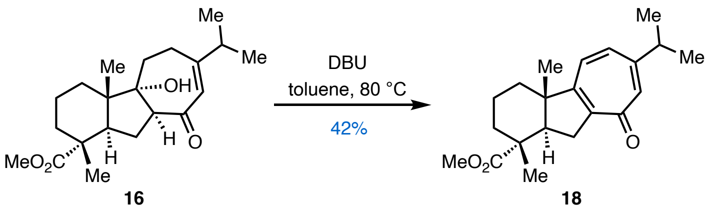

In a flame dried microwave vial containing alcohol **16** (5 mg, 0.014 mmol) under argon atmosphere was added toluene (500 μL) at room temperature. After 5 min, DBU (9.2 μL, 0.03 mmol, 2.0 equiv.) was added. The reaction mixture was heated to 80 °C and stirred overnight before it was cooled to room temperature, quenched with aqueous NH_4Cl (400 μL), and extracted with CH_2Cl_2 (3 x 1 mL). Organic layers were washed with brine, dried over anhydrous Na_2SO_4, filtered and concentrated.

Residue was purified via flash column chromatography (hexanes/ethyl acetate, 4:1 to 1:1) to afford **18** as a clear oil (2.2 mg, 42%)

^{1}H NMR (400 MHz, CDCl$_3$) δ 7.03 (s, 1H), 6.93 (d, J = 11.3 Hz, 1H), 6.83 (d, J = 11.3 Hz, 1H), 3.67 (s, 3H), 2.81 – 2.64 (m, 3H), 2.18 (dd, J = 12.3, 6.5 Hz, 1H), 1.95 (m, 1H), 1.86 – 1.79 (m, 3H), 1.69 (m, 1H), 1.56 (m, 1H), 1.34 (s, 3H), 1.21 (d, J = 6.9 Hz, 6H), 1.08 (m, 3H);

^{13}C NMR (101 MHz, CDCl$_3$) δ 185.3, 178.0, 159.1, 155.8, 153.0, 138.4, 135.9, 129.5, 52.2, 51.2, 49.7, 45.2, 38.7, 35.8, 35.0, 32.3, 23.2, 23.1, 20.5, 19.1, 16.6;

HRMS (APCI): *m/z* calc for $C_{21}H_{29}O_3^+$ $[M+H]^+$: 329.2111, found 329.2111;

IR (film): 2925, 2869, 1724, 1617, 1562, 1474, 1364, 1249, 1173, 1125, 1103, 1056, cm^{-1}.

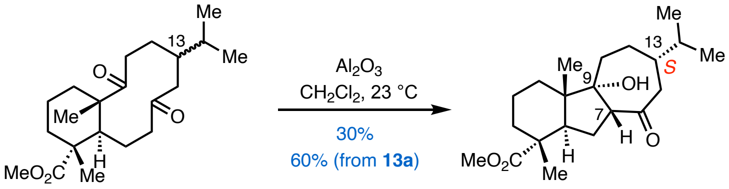

13a (13*S*) & **13b** (13*R*) **19** (13*S*)

A mixture of the diketones **13a/13b** (98 mg, 1:1, 0.28 mmol) and basic alumina (1.0 g) in dichloromethane (10 mL) was stirred at room temperature under argon for 16 h. The alumina was removed by filtration and washed with several portions of dichloromethane. The combined filtrates were concentrated under reduced pressure. The resulting oil was purified via flash column chromatography (hexanes/ethyl acetate, 4:1 to 1:1) to give a clear oil which was crystallized to give **19** as a white solid (29.5 mg, 30%; 60% from **13a**). Note: **13b** decomposed under the conditions.

^{1}H NMR (400 MHz, CDCl$_3$) δ 3.69 (s, 3H), 3.38 (dd, J = 10.6, 3.4 Hz, 1H), 2.40 (ddd, J = 18.6, 3.3, 1.9 Hz, 1H), 2.33 – 2.18 (m, 3H), 1.82 – 1.70 (m, 4H), 1.69 – 1.58 (m, 5H), 1.53 (m, 1H), 1.47 – 1.38 (m, 2H), 1.31 – 1.25 (m, 2H), 1.22 (s, 3H), 0.93 (s, 4H), 0.88 (d, J = 6.6 Hz, 3H), 0.86 (d, J = 6.6 Hz, 3H);

^{13}C NMR (101 MHz, $CDCl_3$) δ 211.3, 178.5, 84.0, 55.8, 52.2, 49.9, 47.0, 45.9, 45.3, 40.7, 35.7, 34.6, 34.2, 30.0, 28.0, 22.5, 19.1, 18.8, 18.6, 17.3, 16.9;

HRMS (ESI): *m/z* calc for $C_{21}H_{34}O_4Na^+$ $[M+Na]^+$: 373.2349, found 373.2345;

IR (film): 2950, 2923, 1726, 1663, 1455, 1385, 1293, 1248, 1183, 1108, 10544, 938, 856, 838, 753 cm^{-1}.

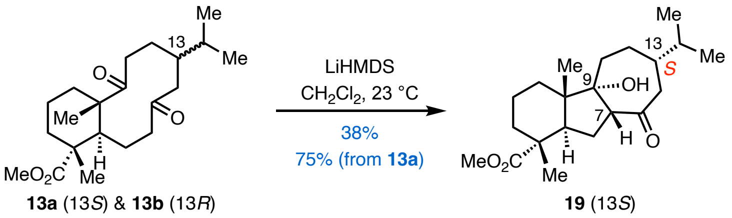

To a flamed-dried vial containing a mixture (1:1) of **13a/13b** (95 mg, 0.27 mmol) was added CH_2Cl_2 (3 mL). The solution was stirred at 0 °C for 5 min, then lithium bis(trimethylsilyl)amide (1 M in THF, 542 µL, 542 µmol, 2 equiv.) was added dropwise. The reaction was warmed to 23 °C and stirred for 16 h. The reaction mixture was then placed at 0 °C and stirred for 5 min before it was quenched with saturated NH_4Cl (1 mL) and extracted with CH_2Cl_2 (3 x 5 mL). The combined organic layers were washed with brine, dried over anhydrous Na_2SO_4, filtered, and concentrated. The resulting pale yellow-brown oil was purified via flash column chromatography (hexanes/ethyl acetate, 4:1 to 1:1) to give a white solid **19** (35.5 mg, 38%; 75% from **13a**).

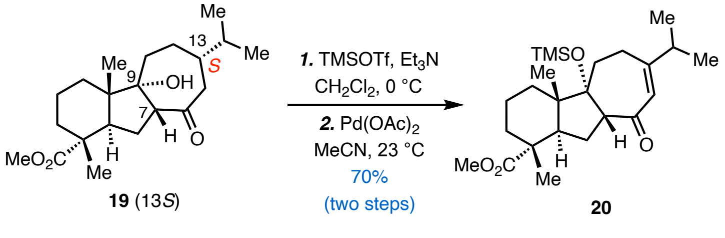

To a flamed-dried vial containing alcohol **19** (61.5 mg, 0.175 mmol) under argon atmosphere was added CH_2Cl_2 (3.5 mL). The solution was stirred at 0 °C for 5 min before triethylamine (978 µL, 7.02 mmol, 40.0 equiv.) was added followed by TMSOTf (637 µL, 3.51 mmol, 20.0 equiv.) dropwise. The reaction mixture was stirred for 1 h at 0 °C before it was quenched with sodium

bicarbonate (1.5 mL) and extracted with CH_2Cl_2 (3 x 2 mL). The combined organic layers were dried over anhydrous Na_2SO_4, filtered and concentrated to give a yellow color oil which was used in the next step without further purification.

The above crude silyl enol ether intermediate was flushed with argon and dissolved in MeCN (3.5 mL). $Pd(OAc)_2$ (43.4 mg, 0.193 mmol) was quickly added. The reaction was stirred overnight at room temperature. The reaction mixture was then filtered through a pad of celite, washed with EtOAc, concentrated, and purified via flash column chromatography (hexanes/ethyl acetate, 4:1 to 1:1) to yield a clear oil **20** (52 mg, 70% over two steps).

^{1}H NMR (400 MHz, $CDCl_3$) δ 5.97 (s, 1H), 3.67 (s, 3H), 3.20 (dd, J = 10.5, 5.5 Hz, 1H), 2.51 (dd, J = 11.4, 8.9 Hz, 1H), 2.48 – 2.38 (m, 3H), 2.34 – 2.23 (m, 2H), 1.79 – 1.53 (m, 6H), 1.49 – 1.37 (m, 2H), 1.20 (s, 3H), 1.08 (d, J = 6.9 Hz, 6H, 2*Me*), 0.91 (s, 3H), 0.03 (s, 9H);

^{13}C NMR (101 MHz, $CDCl_3$) δ 199.5, 178.6, 164.0, 128.6, 89.1, 58.2, 52.0, 51.3, 46.1, 45.4, 39.1, 35.7, 33.1, 30.9, 30.2, 20.91, 20.90, 20.8, 18.6, 17.4, 16.5, 2.9;

HRMS (ESI): *m/z* calc for $C_{24}H_{40}O_4SiNa^+$ $[M+Na]^+$: 443.2588, found 443.2585;

IR (film): 2950, 2923, 1726, 1663, 1455, 1385, 1293, 1248, 1183, 1108, 1054, 938, 856, 838, 753 cm^{-1}.

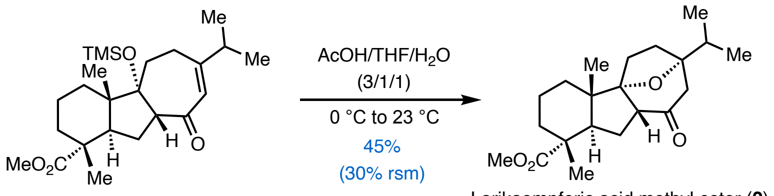

20 Larikaempferic acid methyl ester (**2**)

To a flame-dried vial containing enone **20** (25 mg, 0.059 mmol) under argon atmosphere was added THF (1 mL), distilled water (1 mL) and acetic acid (3 mL). This mixture was stirred at room temperature overnight. After 18 h, saturated $NaHCO_3$ was added. The mixture extracted with ethyl acetate (3 x 4 mL). The combined organic layers were washed with brine and dried over anhydrous Na_2SO_4, filtered, and concentrated. The resulting residue was purified via flash column

chromatography (hexane/ethyl acetate, 6:1 to 4:1) to produce larikaempferic acid methyl ester (**2**) (8 mg, 45%) as a clear oil with 30% recovered of starting material.

^{1}H NMR (400 MHz, $CDCl_3$) δ 3.62 (s, 3H), 2.54 (dd, J = 12.5, 4.1 Hz, 1H), 2.48 (dd, J = 12.5, 8.0 Hz, 1H), 2.42 (d, J = 17.6 Hz, 1H), 2.34 (d, J = 17.6 Hz, 1H), 2.05 (q, J = 12.5 Hz, 1H), 1.95 – 1.82 (m, 2H), 1.80 – 1.69 (m, 3H), 1.69 – 1.63 (m, 3H), 1.63 – 1.59 (m, 2H), 1.55 (m, 1H), 1.35 (m, 1H), 1.19 (s, 3H), 0.90 (d, J = 6.8 Hz, 3H), 0.88 (d, J = 6.8 Hz, 3H), 0.87 (s, 3H);

^{13}C NMR (101 MHz, $CDCl_3$) δ 214.2, 178.6, 92.1, 83.2, 54.5, 52.0, 50.4, 47.6, 46.0, 45.5, 36.4, 35.8, 30.8, 30.6, 29.1, 28.3, 18.3, 17.6, 17.5, 16.7, 16.5;

HRMS (APCI): *m/z* calc for $C_{21}H_{33}O_4$ $^+$[M+H]$^+$: 349.2373, found 349.2376;

IR (film): 2922, 2854, 1726, 1705, 1455, 1384, 1244, 1172, 1136, 1110, 1075, 1026, 938, 860, 801cm^{-1};

$[\alpha]_D^{23}$ = −84.9 (*c* 0.1, $CHCl_3$).

Table 1. ^{1}H and ^{13}C NMR comparison of natural and synthetic larikaempferic acid methyl ester (**2**).

**Position**	**^{1}H NMR δ (ppm), multiplicity, (*J* in Hz)**		**^{13}C NMR δ (ppm)**	
	**Reference[(2)] (500 MHz)**	**Synthetic (400 MHz)**	**Reference[(2)] 125 MHz**	**Synthetic (101 MHz)**
1α	1.73, m	1.73, m	29.0	29.1
1β	1.36, dt (12.0, 3.0)	1.35, m	-	-
2	1.67 (2H), m	1.67 (2H), m	18.1	18.3
3α	1.73, m	1.73, m	35.7	35.8
3β	1.65, m	1.64, m	-	-

4	-	-	45.3	45.5
5α	2.48, dd (12.5, 8.0)	2.48, dd (12.5, 8.0)	47.4	47.6
6α	1.65, m	1.64, m	28.1	28.3
6β	2.04, quartet (12.5)	2.05, quartet (12.5)	-	-
7β	2.55, dd (12.5, 4.5)	2.54, dd (12.5, 4.1)	54.3	54.5
8	-	-	214.0	214.2
9	-	-	92.0	92.1
10	-	-	45.9	46.0
11α	1.73, m	1.73, m	30.4	30.6
11β	1.58, m	1.56, m	-	-
12α	1.73, m	1.73, m	30.6	31.0
12β	1.89, m	1.89, m	-	-
13	-	-	83.0	83.2
14α	2.35, br d (17.0)	2.34, d (17.6)	50.2	50.4
14β	2.42, d (17.0)	2.42, d (17.6)		-
15	1.89, septet (6.5)	1.89, m	36.2	36.4
16	0.89, d (6.5)	0.88, d (6.8)	16.3	16.4
17	0.90, d (6.5)	0.90, d (6.8)	17.3	17.5
18	-	-	178.4	178.6
19	1.20, s	1.19, s	16.5	16.7
20	0.88, s	0.87, s	17.4	17.6
COOMe	3.63, s	3.62, s	51.8	52.0

Part 2. X-ray and Structural Analysis

Compound **14a**

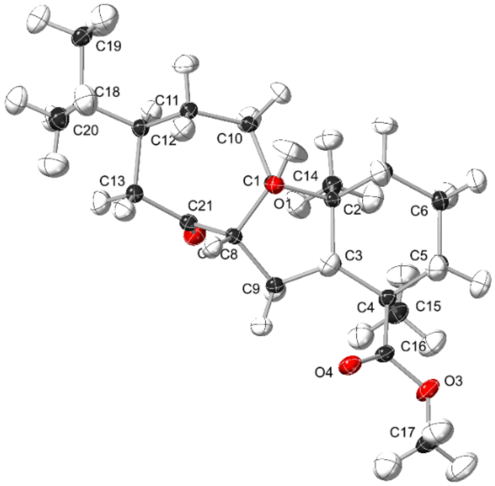

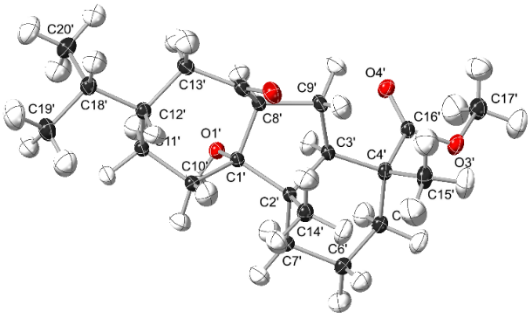

Crystal Data. $C_{21}H_{32}O_4$, M_r = 348.486, orthorhombic, $P2_12_12_1$ (No. 19), a = 9.4597(1) Å, b = 13.7995(1) Å, c = 15.0329(1) Å, $a = b = g = 90^{\circ}$, V = 1962.38(3) Å^3, T = 99.97(16) K, Z = 4, Z' = 1, m(Cu K_a) = 0.637, 20958 reflections measured, 4065 unique (R_{int} = 0.0460) which were used in all calculations. The final wR_2 was 0.0633 (all data) and R_1 was 0.0258 (I≥2 s(I)).

CCDC number	2326256
Formula	$C_{21}H_{34}O_4$
$D_{calc.}$/ g cm^{-3}	1.195
m/mm^{-1}	0.642
Formula Weight	350.502
Color	colorless
Shape	needle-shaped
Size/mm^3	0.30×0.23×0.19
T/K	100.02(10)
Crystal System	monoclinic
Flack Parameter	-0.07(6)
Hooft Parameter	-0.07(6)
Space Group	$I2$
a/Å	20.0209(2)
b/Å	6.9482(1)
c/Å	29.3036(2)
a/°	90
b/°	107.026(1)
g/°	90
V/Å^3	3897.74(8)
Z	8
Z'	2
Wavelength/Å	1.54184
Radiation type	Cu K_a
Q_{min}/°	3.15
Q_{max}/°	72.49
Measured Refl's.	27039
Indep't Refl's	7060
Refl's I≥2 s(I)	6876
R_{int}	0.0421
Parameters	1064
Restraints	1099
Largest Peak	0.1121
Deepest Hole	-0.1056
GooF	1.0912
wR_2 (all data)	0.0605
wR_2	0.0601
R_1 (all data)	0.0258
R_1	0.0251

Compound **14b**

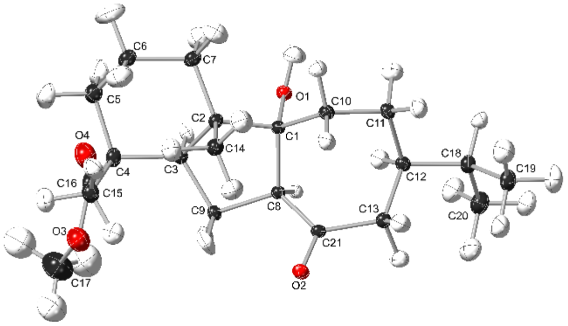

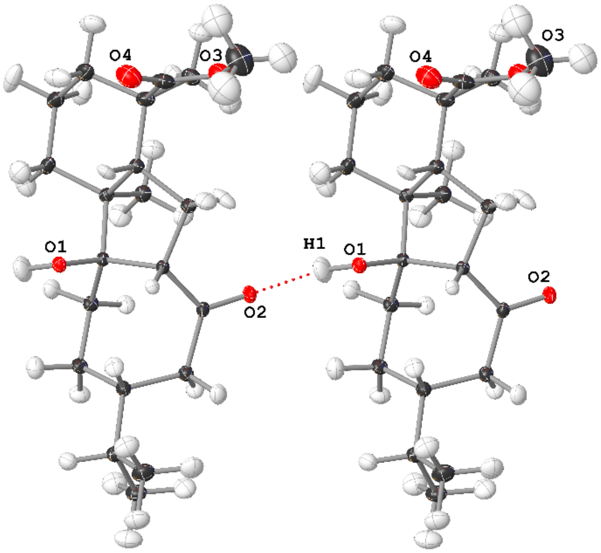

CCDC number	2326255
Empirical formula	$C_{21}H_{34}O_4$
Formula weight	350.502
Temperature [K]	100(2)
Crystal system	orthorhombic
Space group (number)	$P2_12_12_1$ (19)
a [Å]	6.9015(1)
b [Å]	11.1195(1)
c [Å]	24.8315(3)
α [°]	90
β [°]	90
γ [°]	90
Volume [Å^3]	1905.60(4)
Z	4
ρ_{calc} [gcm^{-3}]	1.222
μ [mm^{-1}]	0.656
F(000)	770.451
Crystal size [mm^3]	0.23×0.35×0.37
Crystal colour	colorless
Crystal shape	prism
Radiation	Cu K_α (λ=1.54178 Å)
2θ range [°]	7.12 to 147.40 (0.80 Å)
Index ranges	$-8 \le h \le 8$<br>$-13 \le k \le 13$<br>$-30 \le l \le 18$
Reflections collected	20285
Independent reflections	3712<br>R_{int} = 0.0306<br>R_{sigma} = 0.0170
Completeness to θ = 67.6840°	99.1 %
Data / Restraints / Parameters	3712 / 600 / 683
Goodness-of-fit on F^2	1.1437
Final R indexes [$I \geq 2\sigma(I)$]	R_1 = 0.0170<br>wR_2 = 0.0433
Final R indexes [all data]	R_1 = 0.0171<br>wR_2 = 0.0434
Largest peak/hole [eÅ^{-3}]	0.12/−0.08
Extinction coefficient	0.0011
Flack X parameter	0.02(4)

Compound **19**

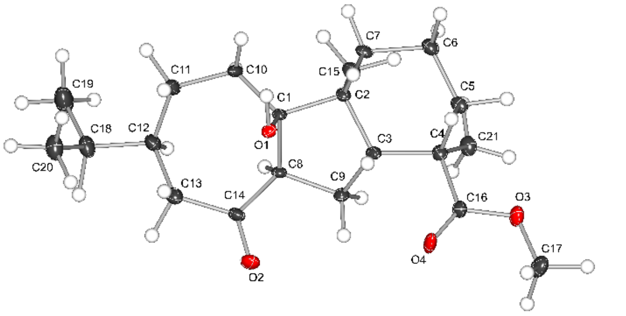

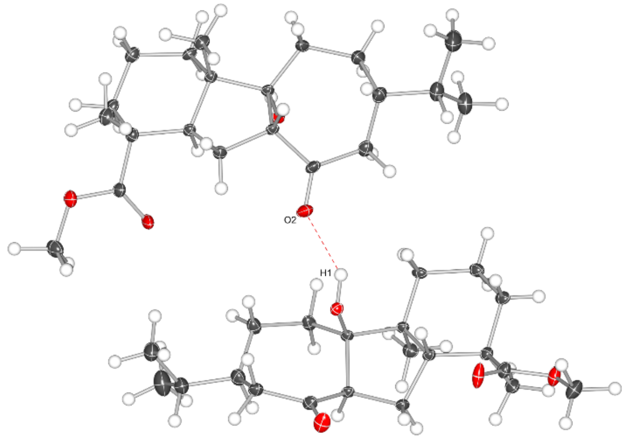

CCDC number	2326254
Empirical formula	$C_{42}H_{68}O_8$
Formula weight	701.003
Temperature/K	105(8)
Crystal system	monoclinic
Space group	$P2_1$
a/Å	6.1621(4)
b/Å	11.6465(6)
c/Å	13.7206(9)
α/°	90
β/°	102.517(6)
γ/°	90
Volume/Å^3	961.28(10)
Z	1
ρ_{calc}g/cm^3	1.211
μ/mm^{-1}	0.650
F(000)	385.2
Crystal size/mm^3	0.28 × 0.08 × 0.07
Radiation	Cu Kα (λ = 1.54184)
2Θ range for data collection/°	10.06 to 145.94
Index ranges	$-7 \le h \le 7$<br>$-13 \le k \le 13$<br>$-16 \le l \le 16$
Reflections collected	11114
Independent reflections	3443<br>[R_{int} = 0.0552<br>R_{sigma} = 0.0494]
Data/restraints/parameters	3443/867/405
Goodness-of-fit on F^2	1.055
Final R indexes [I>=2σ (I)]	R_1 = 0.0649<br>wR_2 = 0.1264
Final R indexes [all data]	R_1 = 0.0657<br>wR_2 = 0.1268
Largest diff. peak/hole / e Å^{-3}	0.37/-0.39
Flack parameter	-0.26(13)

Part 3. References

1. A. Abad, M. Arnó, L.R. Domingo, R.J. Zaragozá. *Tetrahedron*, 1985, **41**, 4937−4940.
2. H. Ohtsu, R. Tanaka, S. Matsunaga, H. Tokuda, H. Nishino, *Planta Med.*, 1999, **65**, 664−666.

Part 4. ^{1}H and ^{13}C NMR

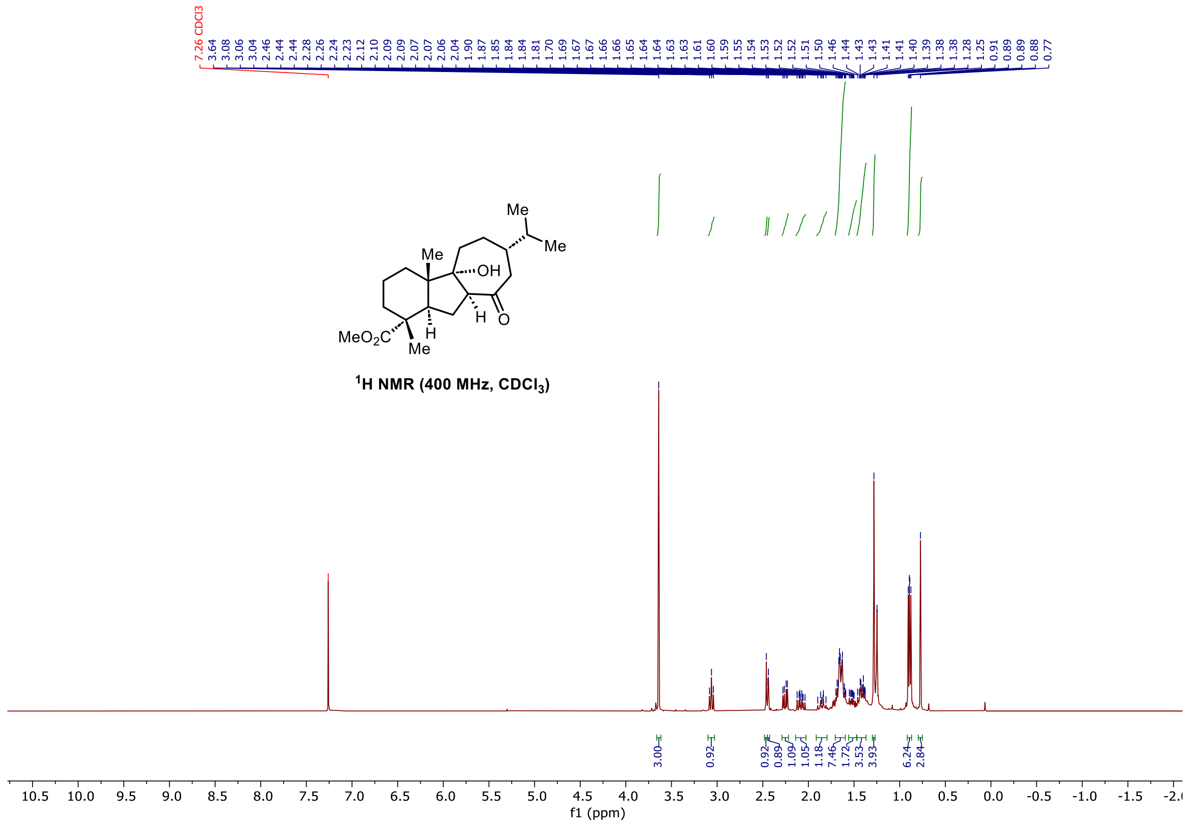

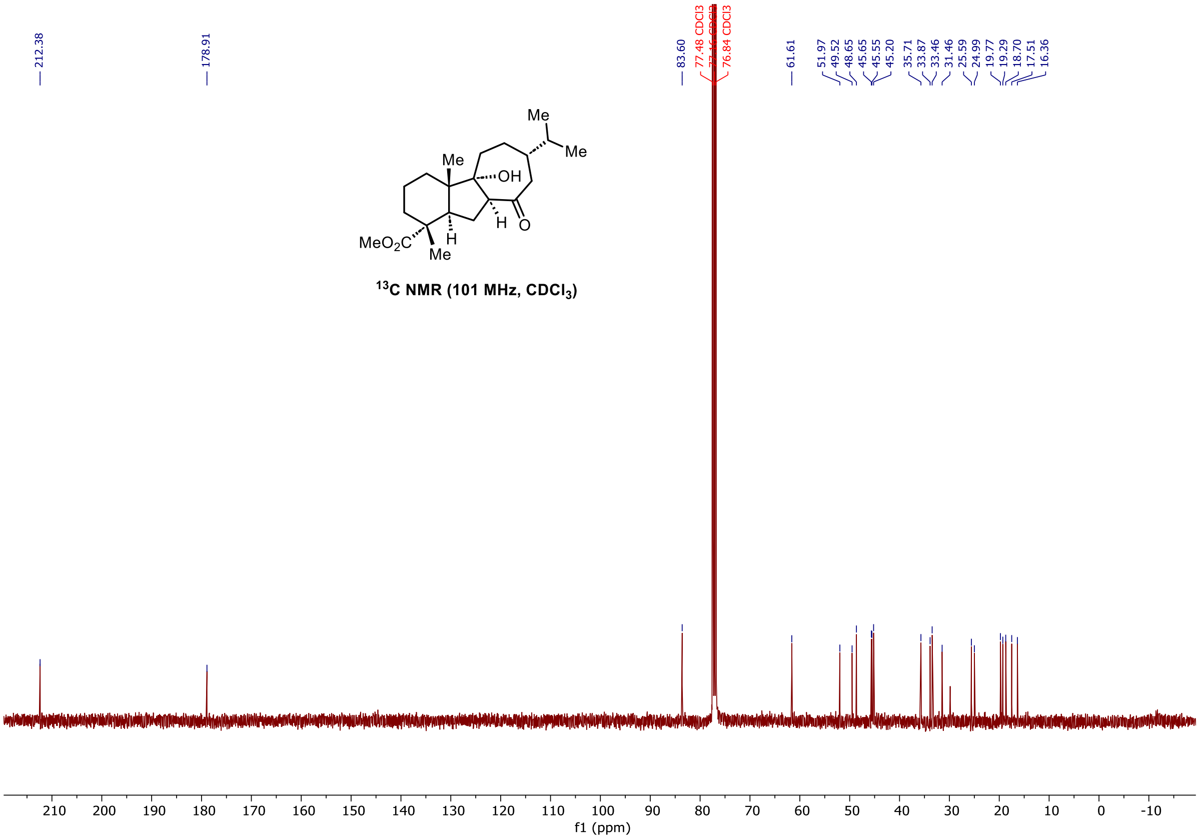

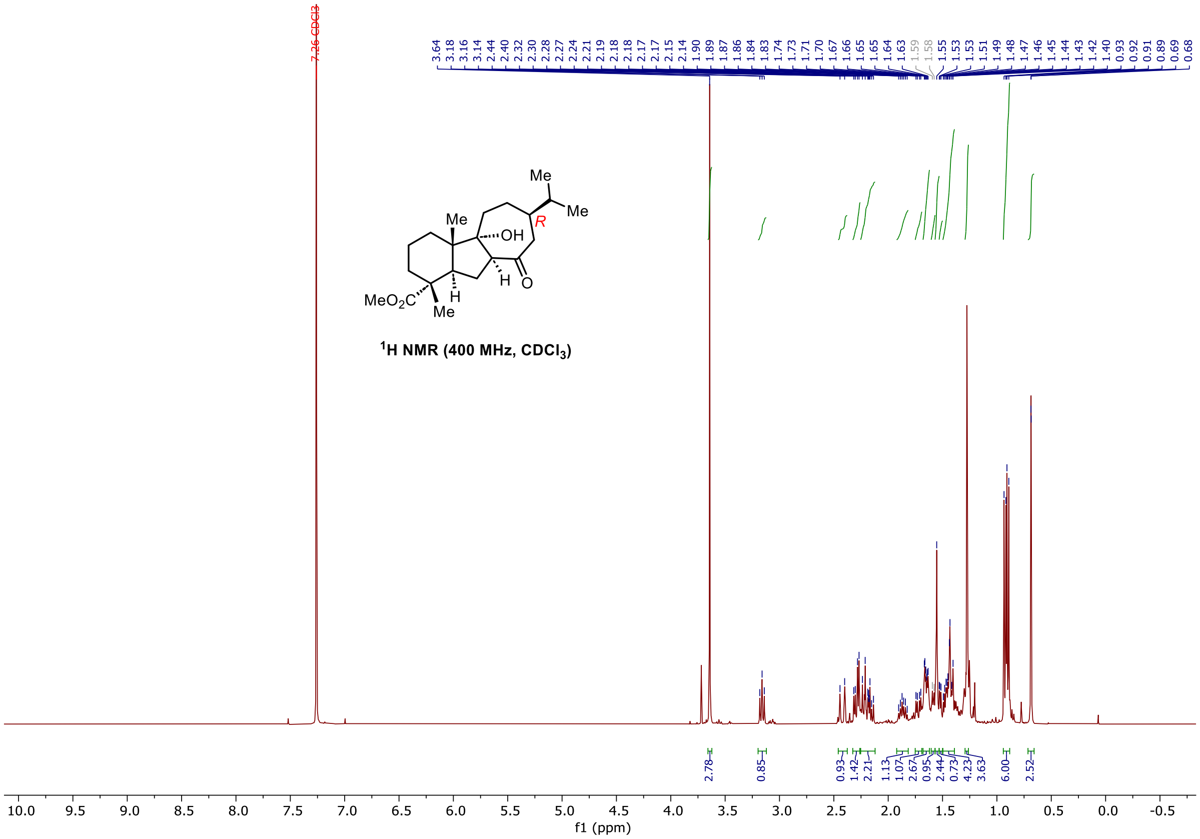
7.26 CDCl3
3.64
3.18
3.16
3.14
2.44
2.40
2.32
2.30
2.28
2.27
2.24
2.21
2.19
2.18
2.18
2.17
2.17
2.15
2.14
1.90
1.89
1.87
1.86
1.84
1.83
1.74
1.73
1.71
1.70
1.67
1.66
1.65
1.65
1.64
1.63
1.59
1.58
1.55
1.53
1.53
1.51
1.49
1.48
1.47
1.46
1.45
1.44
1.43
1.42
1.40
0.93
0.92
0.91
0.89
0.69
0.68
Me
Me
Me
OH
R
H
O
MeO2C
H
Me
1H NMR (400 MHz, CDCl3)
2.78
0.85
0.93
1.42
2.21
1.13
1.07
2.67
0.95
2.44
0.73
4.23
3.63
6.00
2.52
10.0
9.5
9.0
8.5
8.0
7.5
7.0
6.5
6.0
5.5
5.0
4.5
4.0
3.5
3.0
2.5
2.0
1.5
1.0
0.5
0.0
-0.5
f1 (ppm)

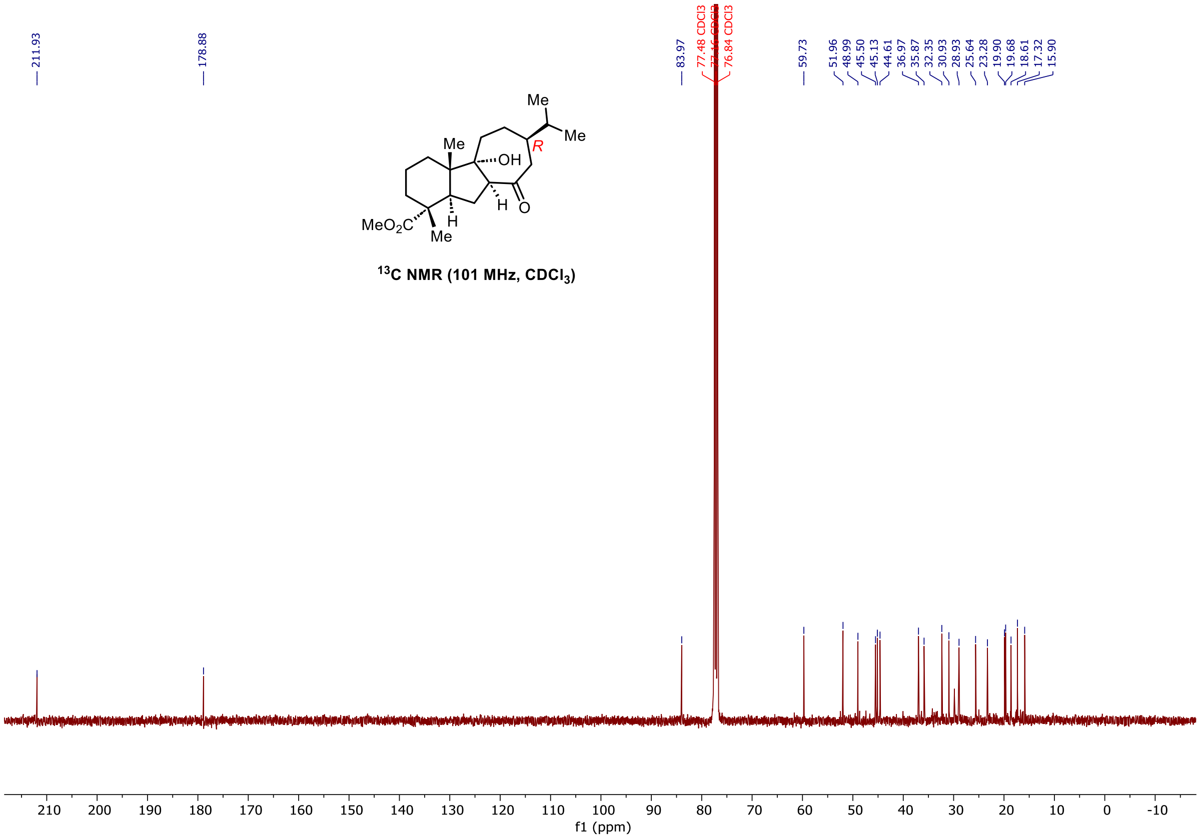
211.93
178.88
83.97
77.48 CDCl3
77.16 CDCl3
76.84 CDCl3
59.73
51.96
48.99
45.50
45.13
44.61
36.97
35.87
32.35
30.93
28.93
25.64
23.28
19.90
19.68
18.61
17.32
15.90
Me
Me
Me
OH
R
H
O
MeO2C
H
Me
13C NMR (101 MHz, CDCl3)
210
200
190
180
170
160
150
140
130
120
110
100
90
80
70
60
50
40
30
20
10
0
-10
f1 (ppm)

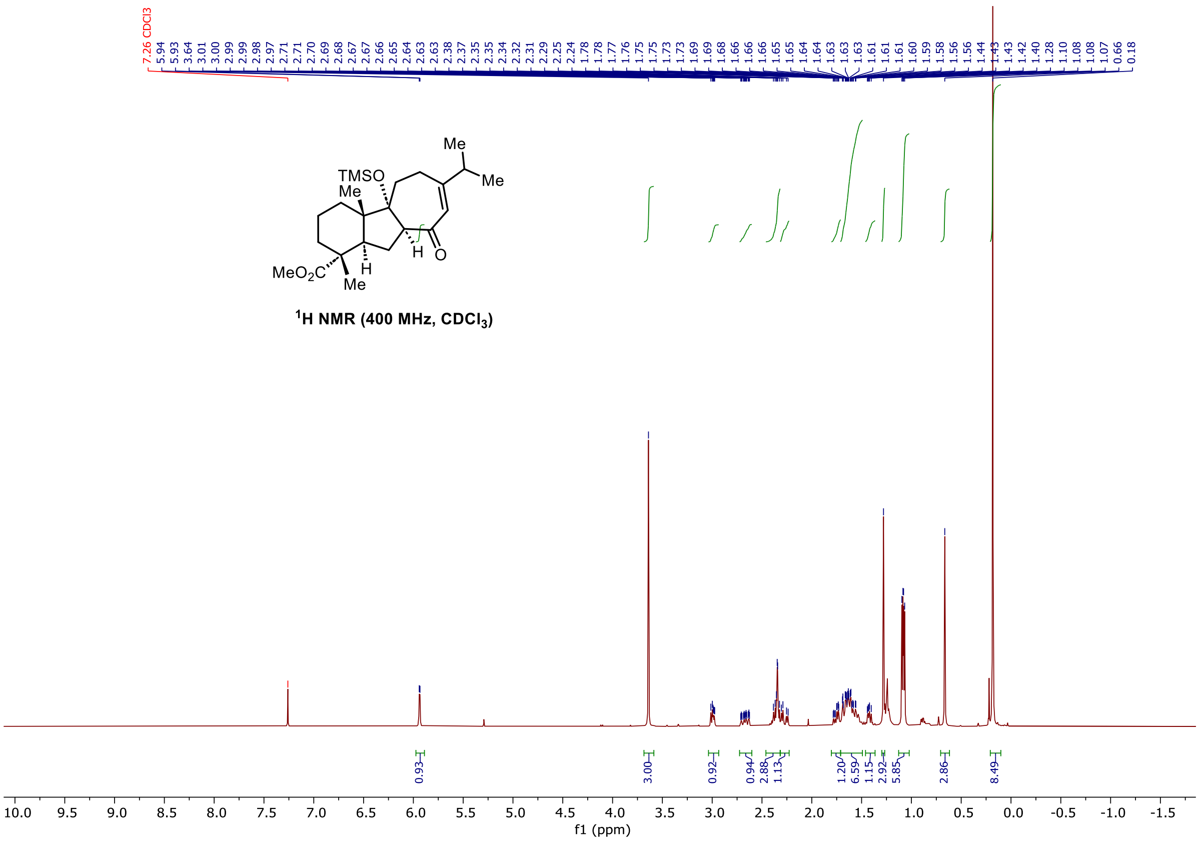
7.26 CDCl3
5.94 5.93 3.64 3.01 3.00 2.99 2.99 2.98 2.97 2.71 2.71 2.70 2.69 2.68 2.67 2.67 2.66 2.65 2.64 2.63 2.63 2.38 2.37 2.35 2.35 2.34 2.32 2.31 2.29 2.25 2.24 1.78 1.78 1.77 1.76 1.75 1.75 1.73 1.73 1.69 1.69 1.68 1.66 1.66 1.66 1.65 1.65 1.64 1.64 1.63 1.63 1.63 1.61 1.61 1.60 1.60 1.59 1.58 1.56 1.56 1.44 1.43 1.43 1.42 1.40 1.28 1.10 1.08 1.08 1.07 0.66 0.18
TMSO
Me
Me
Me
H
H
O
MeO2C
Me
1H NMR (400 MHz, CDCl3)
0.93
3.00
0.92
0.94
2.88
1.13
1.20
6.59
1.15
2.92
5.85
2.86
8.49
f1 (ppm)
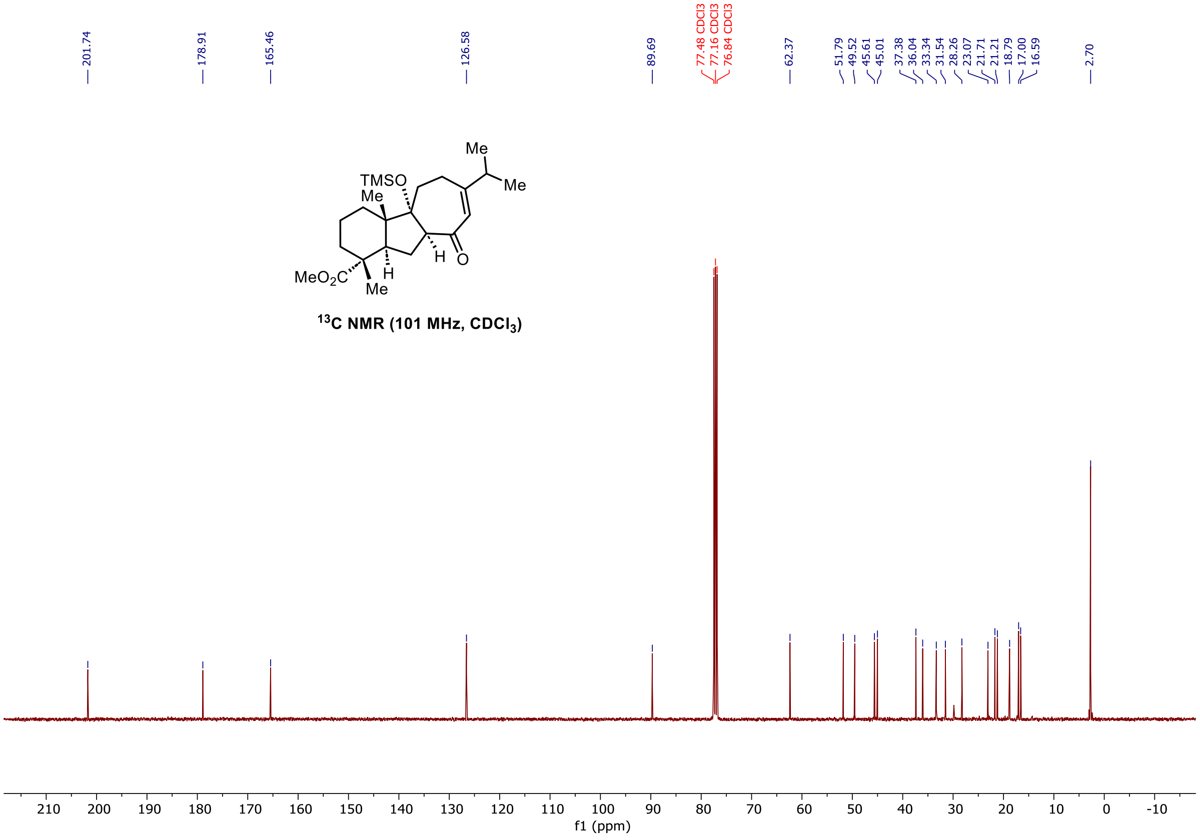
201.74
178.91
165.46
126.58
89.69
77.48 CDCl3
77.16 CDCl3
76.84 CDCl3
62.37
51.79 49.52 45.61 45.01 37.38 36.04 33.34 31.54 28.26 23.07 21.71 21.21 18.79 17.00 16.59
2.70
TMSO
Me
Me
Me
H
H
O
MeO2C
Me
13C NMR (101 MHz, CDCl3)
f1 (ppm)

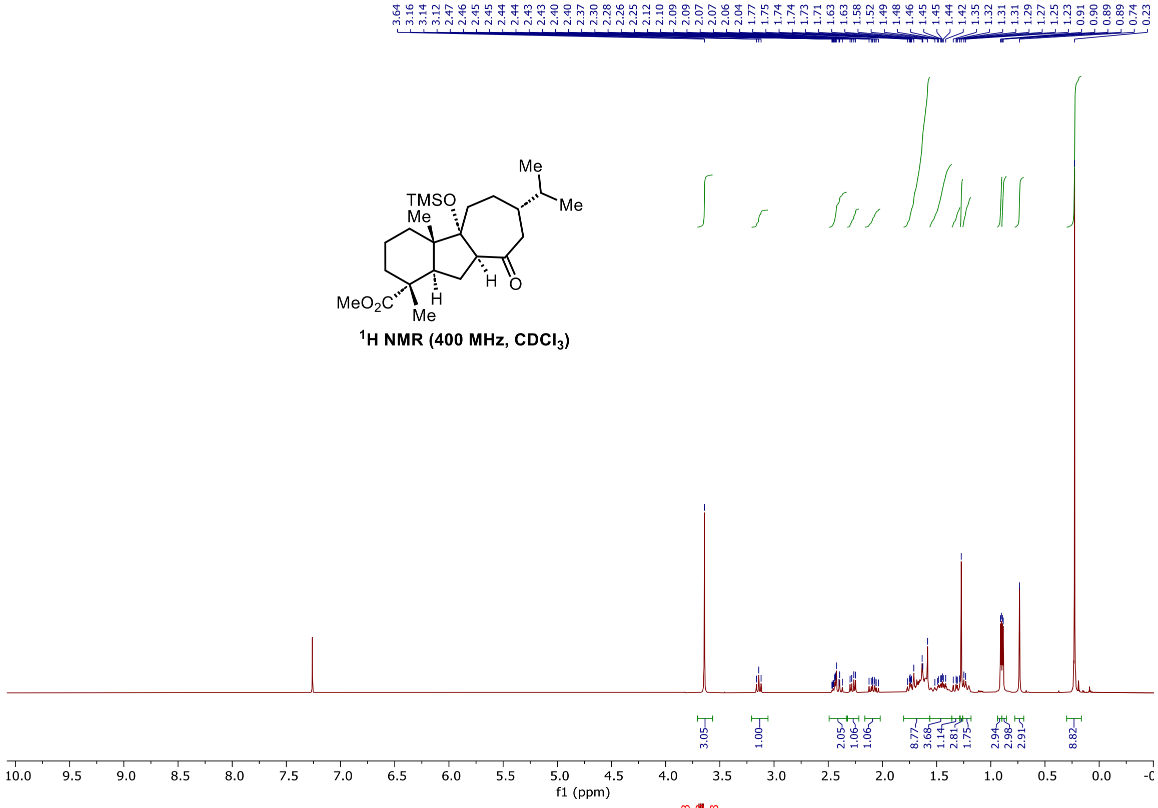

^{1}H NMR (400 MHz, CDCl$_3$)

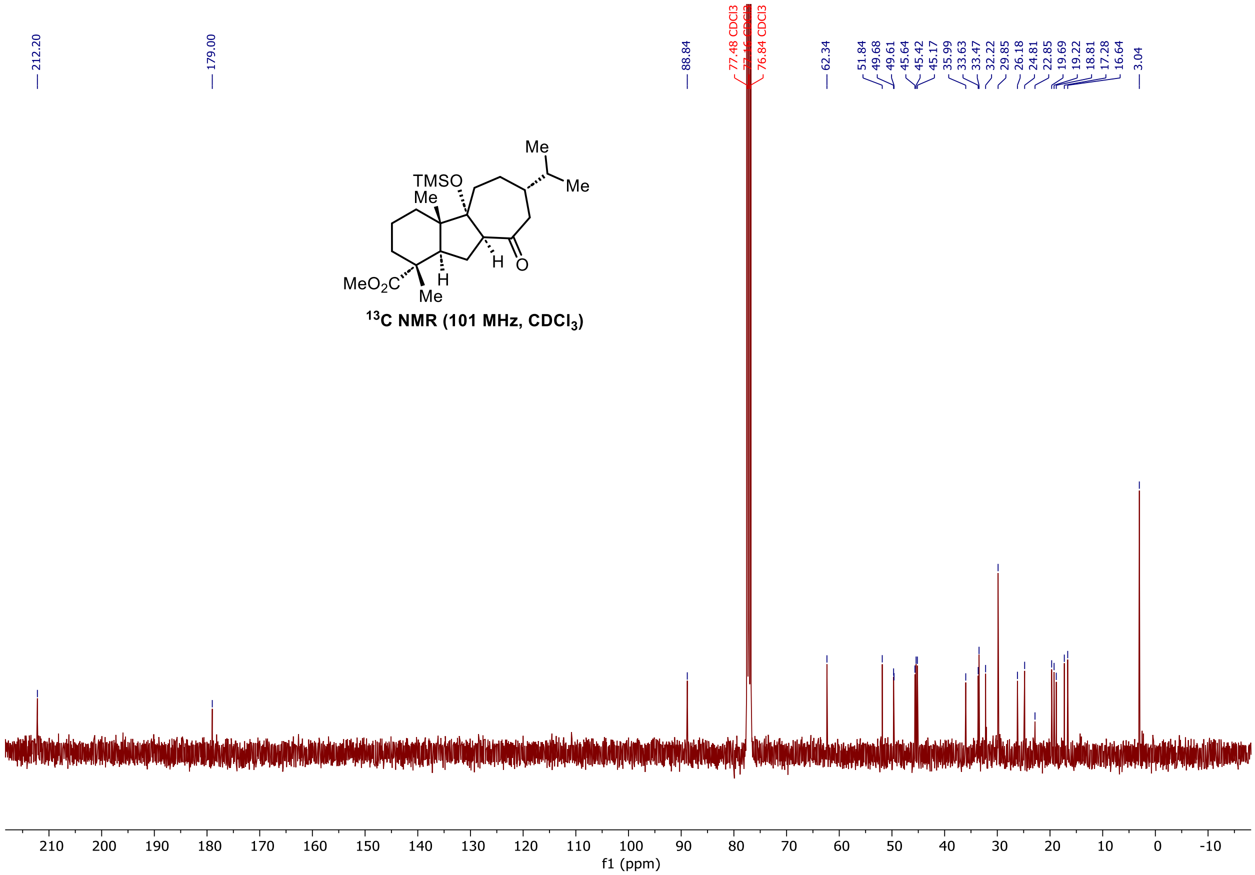

^{13}C NMR (101 MHz, CDCl$_3$)

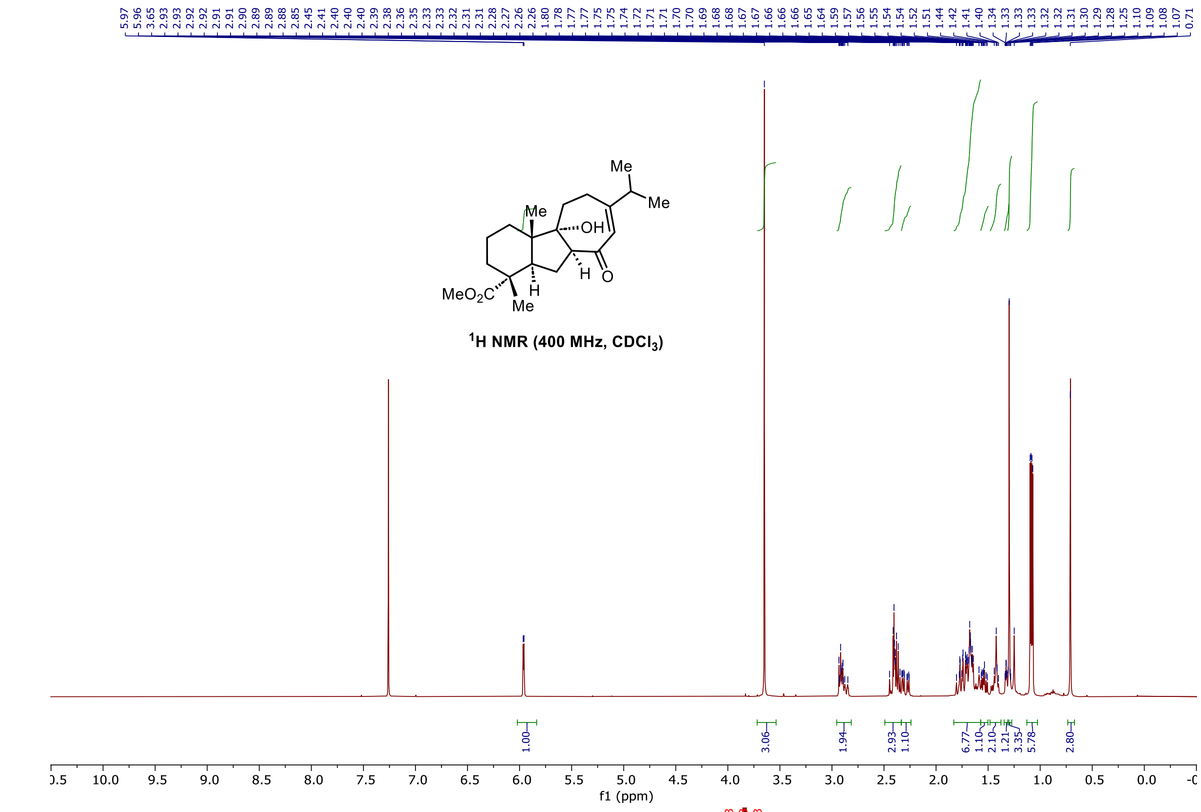
5.97 5.96 3.65 2.93 2.93 2.92 2.92 2.91 2.91 2.90 2.89 2.89 2.88 2.85 2.45 2.41 2.40 2.40 2.39 2.38 2.36 2.35 2.33 2.33 2.32 2.31 2.31 2.28 2.27 2.26 2.26 1.80 1.78 1.77 1.77 1.75 1.75 1.74 1.72 1.71 1.71 1.70 1.69 1.68 1.68 1.67 1.67 1.66 1.66 1.66 1.65 1.64 1.59 1.57 1.56 1.55 1.54 1.54 1.52 1.51 1.44 1.42 1.41 1.40 1.34 1.33 1.33 1.33 1.32 1.32 1.31 1.30 1.29 1.28 1.25 1.10 1.09 1.08 1.07 0.71
Me
Me
Me
OH
H
O
MeO2C
H
Me
1H NMR (400 MHz, CDCl3)
1.00
3.06
1.94
2.93
1.10
6.77
1.10
2.10
1.21
3.35
5.78
2.80
10.0 9.5 9.0 8.5 8.0 7.5 7.0 6.5 6.0 5.5 5.0 4.5 4.0 3.5 3.0 2.5 2.0 1.5 1.0 0.5 0.0
f1 (ppm)

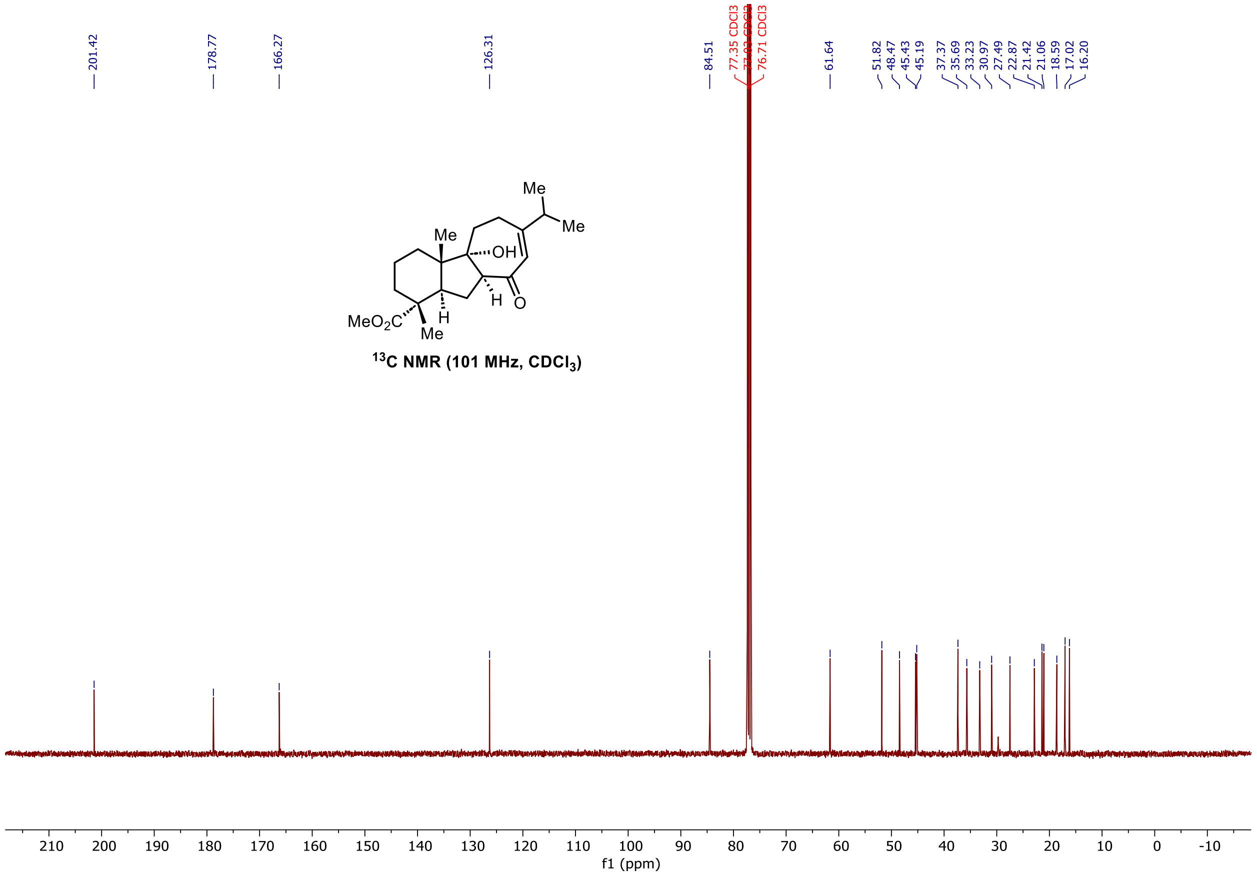
201.42
178.77
166.27
126.31
84.51
77.35 CDCl3
77.03 CDCl3
76.71 CDCl3
61.64
51.82 48.47 45.43 45.19
37.37 35.69 33.23 30.97 27.49 22.87 21.42 21.06 18.59 17.02 16.20
Me
Me
Me
OH
H
O
MeO2C
H
Me
13C NMR (101 MHz, CDCl3)
210 200 190 180 170 160 150 140 130 120 110 100 90 80 70 60 50 40 30 20 10 0 -10
f1 (ppm)

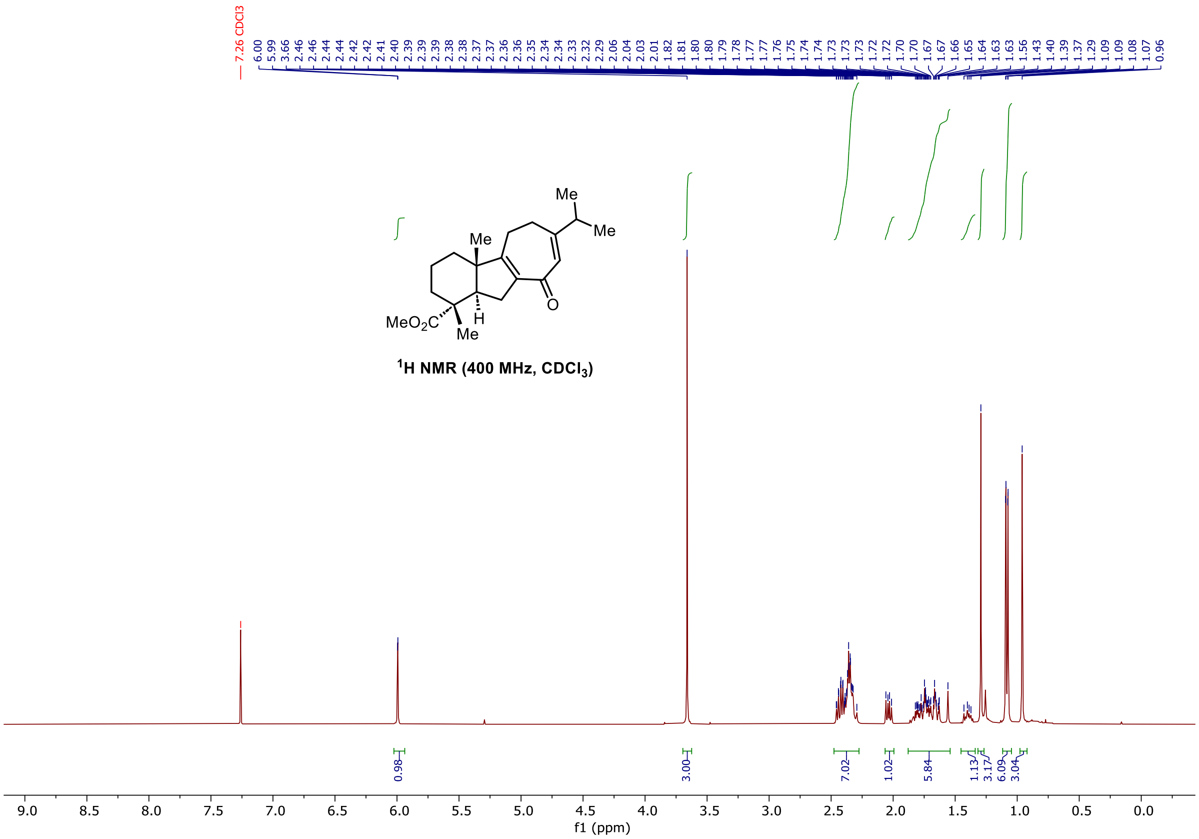
7.26 CDCl3
6.00 5.99 3.66 2.46 2.46 2.44 2.44 2.42 2.42 2.41 2.40 2.39 2.39 2.39 2.39 2.38 2.38 2.37 2.37 2.36 2.36 2.35 2.34 2.34 2.33 2.32 2.29 2.06 2.04 2.03 2.01 1.82 1.81 1.80 1.80 1.79 1.78 1.77 1.77 1.76 1.75 1.74 1.74 1.73 1.73 1.73 1.73 1.72 1.72 1.70 1.67 1.67 1.66 1.65 1.64 1.63 1.63 1.56 1.43 1.40 1.39 1.37 1.29 1.09 1.09 1.08 1.07 0.96
Me
Me
Me
MeO2C
H
O
Me
1H NMR (400 MHz, CDCl3)
0.98
3.00
7.02
1.02
5.84
1.13
3.17
6.09
3.04
9.0 8.5 8.0 7.5 7.0 6.5 6.0 5.5 5.0 4.5 4.0 3.5 3.0 2.5 2.0 1.5 1.0 0.5 0.0
f1 (ppm)

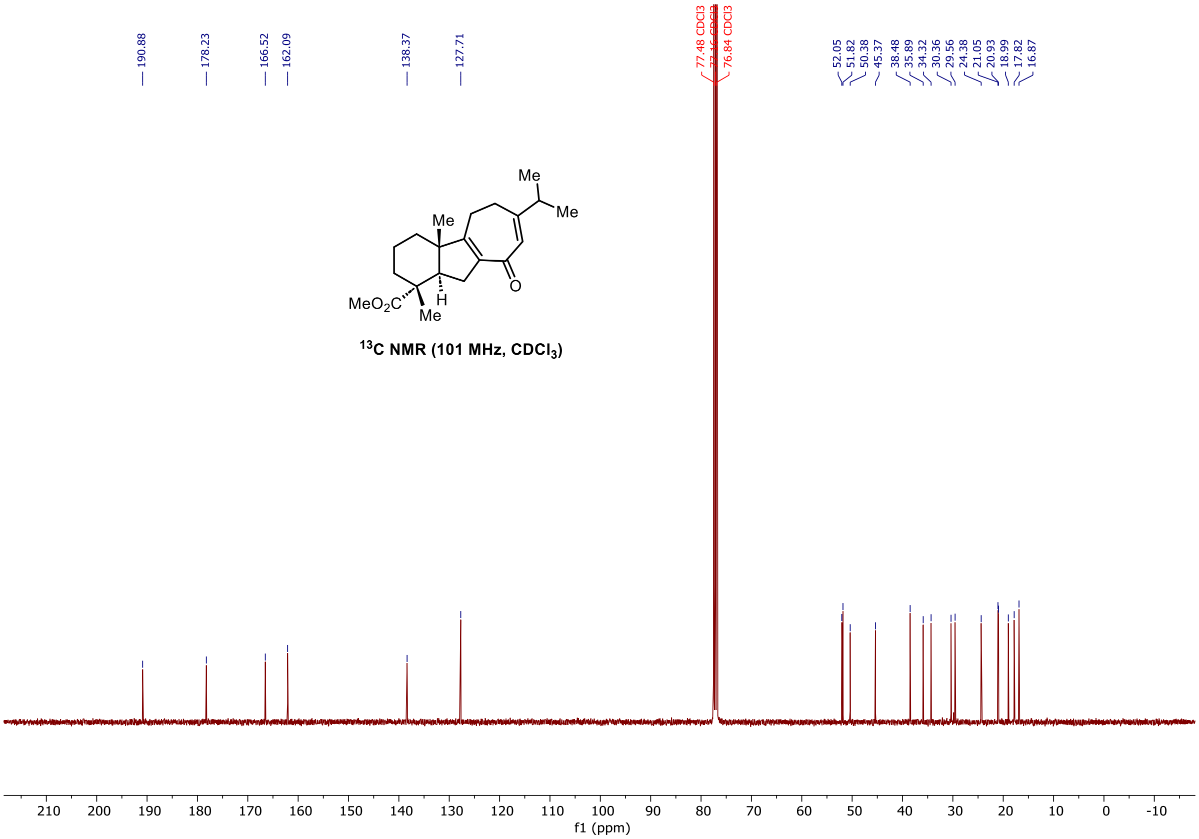
190.88
178.23
166.52
162.09
138.37
127.71
77.48 CDCl3
77.16 CDCl3
76.84 CDCl3
52.05 51.82 50.38 45.37 38.48 35.89 34.32 30.36 29.56 24.38 21.05 20.93 18.99 17.82 16.87
Me
Me
Me
MeO2C
H
O
Me
13C NMR (101 MHz, CDCl3)
210 200 190 180 170 160 150 140 130 120 110 100 90 80 70 60 50 40 30 20 10 0 -10
f1 (ppm)

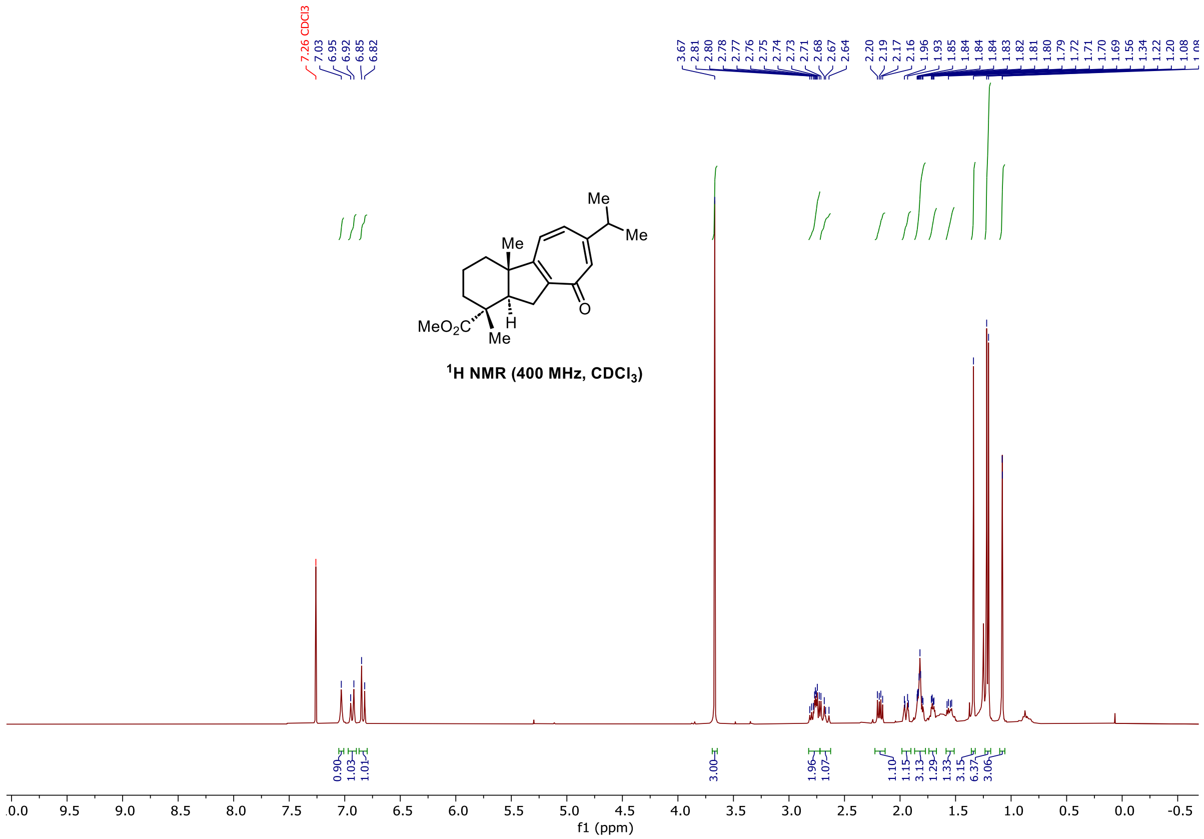
7.26 CDCl3
7.03
6.95
6.92
6.85
6.82
3.67
2.81
2.80
2.78
2.77
2.76
2.75
2.74
2.73
2.71
2.68
2.67
2.64
2.20
2.19
2.17
2.16
1.96
1.93
1.85
1.84
1.84
1.84
1.83
1.82
1.81
1.80
1.79
1.72
1.71
1.70
1.69
1.56
1.34
1.22
1.20
1.08
Me
Me
Me
MeO2C
H
O
Me
1H NMR (400 MHz, CDCl3)
0.90
1.03
1.01
3.00
1.96
1.07
1.10
1.15
3.13
1.29
1.33
3.15
6.37
3.06
f1 (ppm)

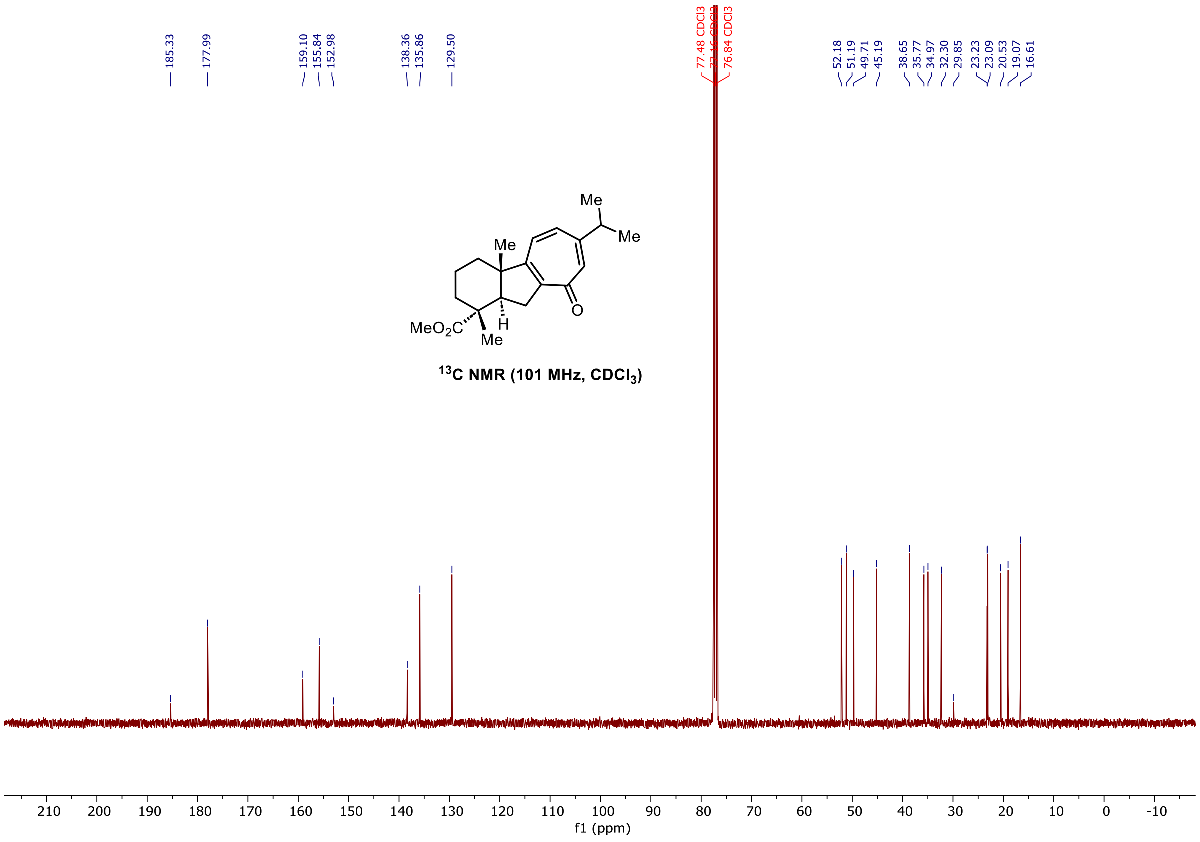
185.33
177.99
159.10
155.84
152.98
138.36
135.86
129.50
77.48 CDCl3
76.84 CDCl3
52.18
51.19
49.71
45.19
38.65
35.77
34.97
32.30
29.85
23.23
23.09
20.53
19.07
16.61
Me
Me
Me
MeO2C
H
O
Me
13C NMR (101 MHz, CDCl3)
f1 (ppm)

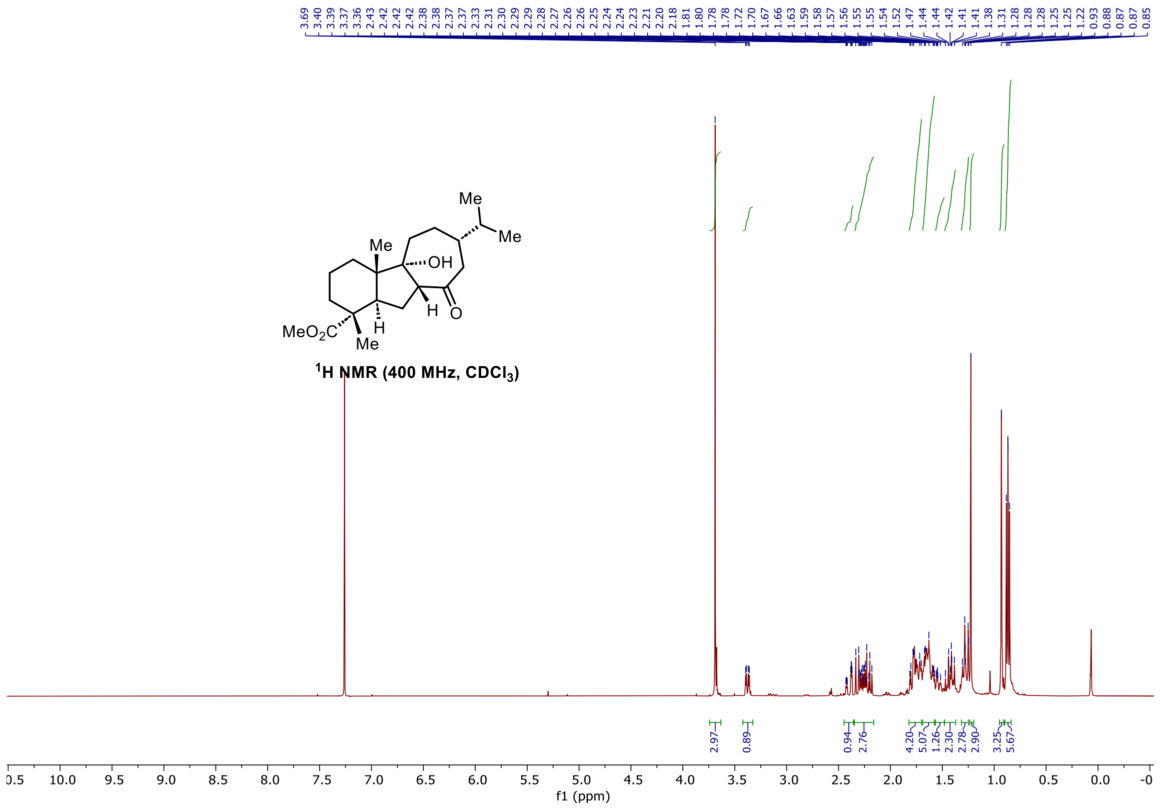
3.69 3.40 3.39 3.37 3.36 2.43 2.42 2.42 2.42 2.38 2.38 2.37 2.37 2.33 2.31 2.30 2.29 2.29 2.28 2.27 2.26 2.26 2.25 2.24 2.24 2.23 2.21 2.20 2.18 1.81 1.80 1.78 1.78 1.72 1.70 1.67 1.66 1.63 1.59 1.58 1.57 1.56 1.55 1.55 1.54 1.52 1.47 1.44 1.44 1.42 1.41 1.41 1.38 1.31 1.28 1.28 1.28 1.25 1.22 0.93 0.88 0.87 0.85
Me
Me
Me
OH
H
O
MeO2C
H
Me
1H NMR (400 MHz, CDCl3)
2.97 0.89 0.94 2.76 4.20 5.07 1.26 2.30 2.78 2.90 3.25 5.67
10.5 10.0 9.5 9.0 8.5 8.0 7.5 7.0 6.5 6.0 5.5 5.0 4.5 4.0 3.5 3.0 2.5 2.0 1.5 1.0 0.5 0.0 -0
f1 (ppm)

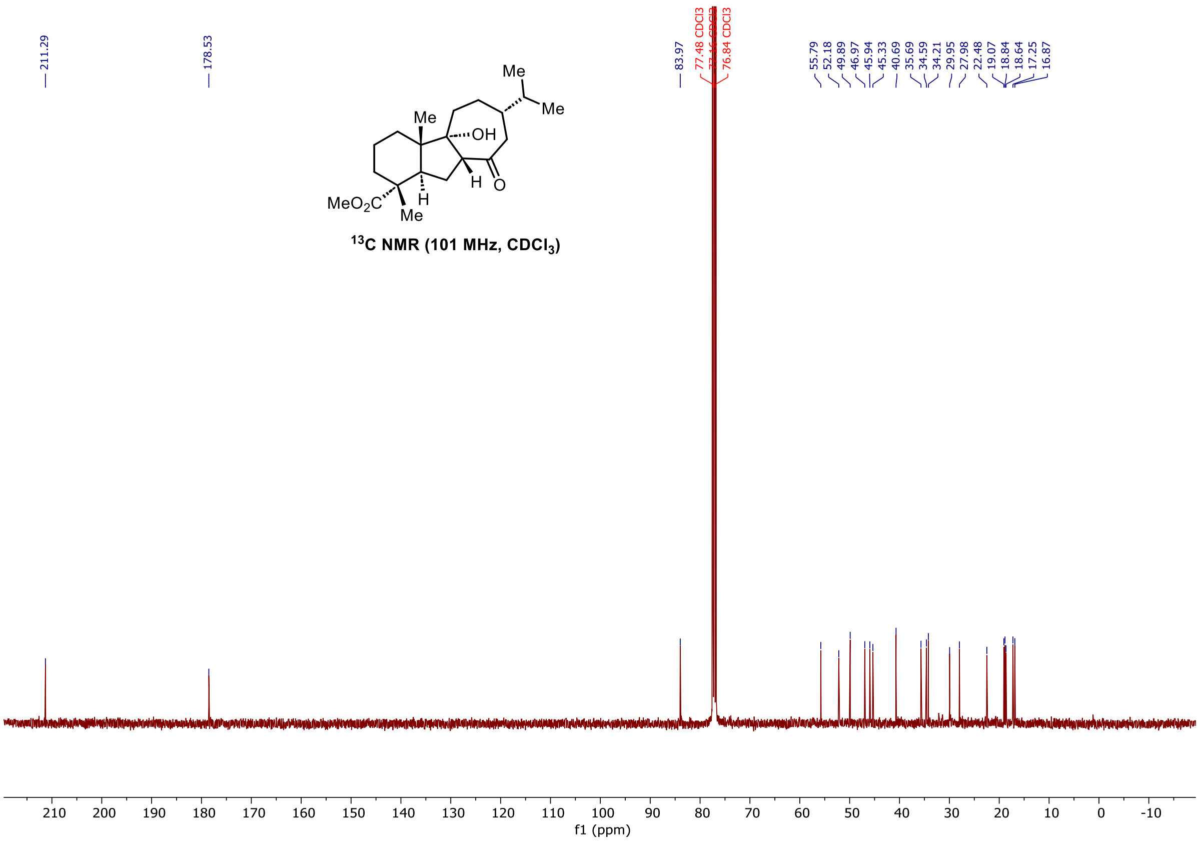
211.29
178.53
83.97
77.48 CDCl3
77.16 CDCl3
76.84 CDCl3
55.79 52.18 49.89 46.97 45.94 45.33 40.69 35.69 34.59 34.21 29.95 27.98 22.48 19.07 18.84 18.64 17.25 16.87
Me
Me
Me
OH
H
O
MeO2C
H
Me
13C NMR (101 MHz, CDCl3)
210 200 190 180 170 160 150 140 130 120 110 100 90 80 70 60 50 40 30 20 10 0 -10
f1 (ppm)

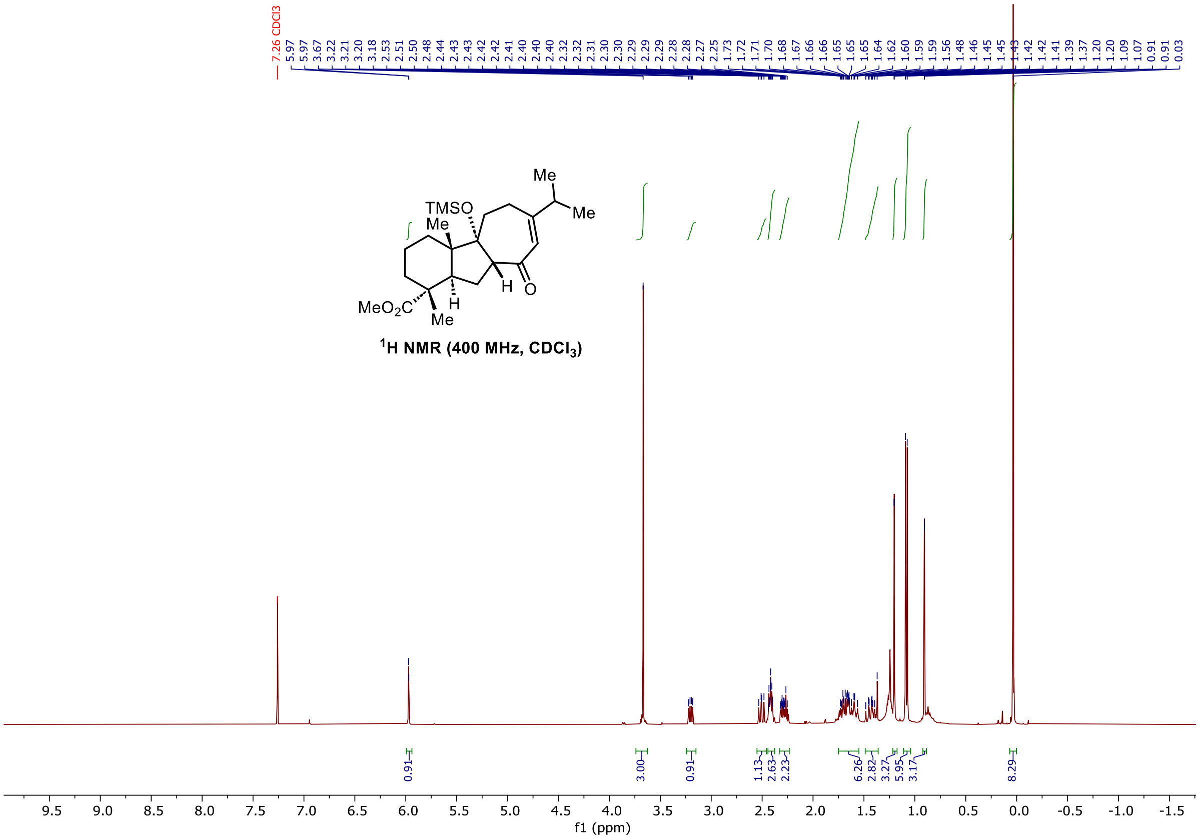
7.26 CDCl3
5.97 5.97 3.67 3.22 3.21 3.20 3.18 2.53 2.51 2.50 2.48 2.44 2.43 2.43 2.42 2.42 2.41 2.40 2.40 2.40 2.32 2.32 2.31 2.30 2.30 2.29 2.29 2.29 2.28 2.27 2.25 1.73 1.72 1.71 1.70 1.68 1.67 1.66 1.66 1.65 1.65 1.65 1.64 1.62 1.60 1.59 1.59 1.56 1.48 1.46 1.45 1.45 1.43 1.42 1.41 1.39 1.37 1.20 1.20 1.09 1.07 0.91 0.91 0.03
TMSO
Me
Me
Me
MeO2C
H
H
O
Me
1H NMR (400 MHz, CDCl3)
0.91 3.00 0.91 1.13 2.63 2.23 6.26 2.82 3.27 5.95 3.17 8.29
9.5 9.0 8.5 8.0 7.5 7.0 6.5 6.0 5.5 5.0 4.5 4.0 3.5 3.0 2.5 2.0 1.5 1.0 0.5 0.0 -0.5 -1.0 -1.5
f1 (ppm)

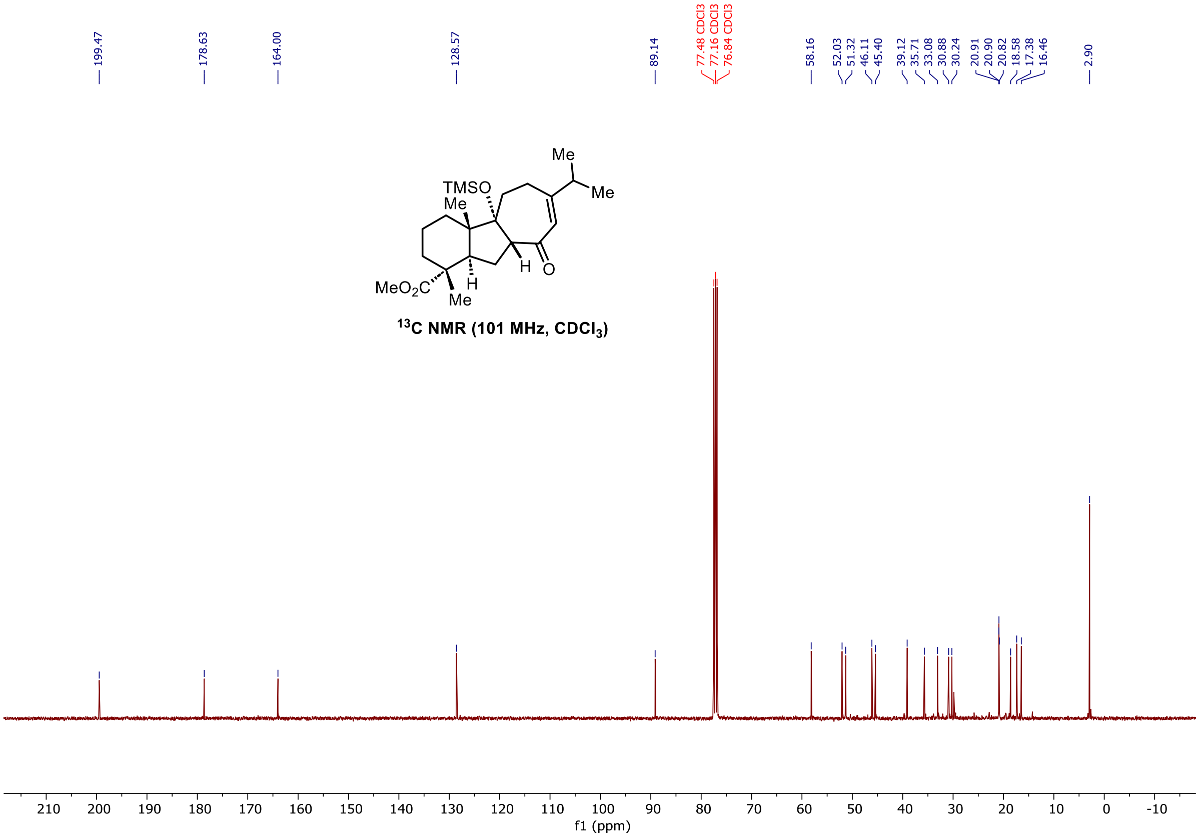
199.47 178.63 164.00 128.57 89.14
77.48 CDCl3 77.16 CDCl3 76.84 CDCl3
58.16 52.03 51.32 46.11 45.40 39.12 35.71 33.08 30.88 30.24 20.91 20.90 20.82 18.58 17.38 16.46 2.90
TMSO
Me
Me
Me
MeO2C
H
H
O
Me
13C NMR (101 MHz, CDCl3)
210 200 190 180 170 160 150 140 130 120 110 100 90 80 70 60 50 40 30 20 10 0 -10
f1 (ppm)

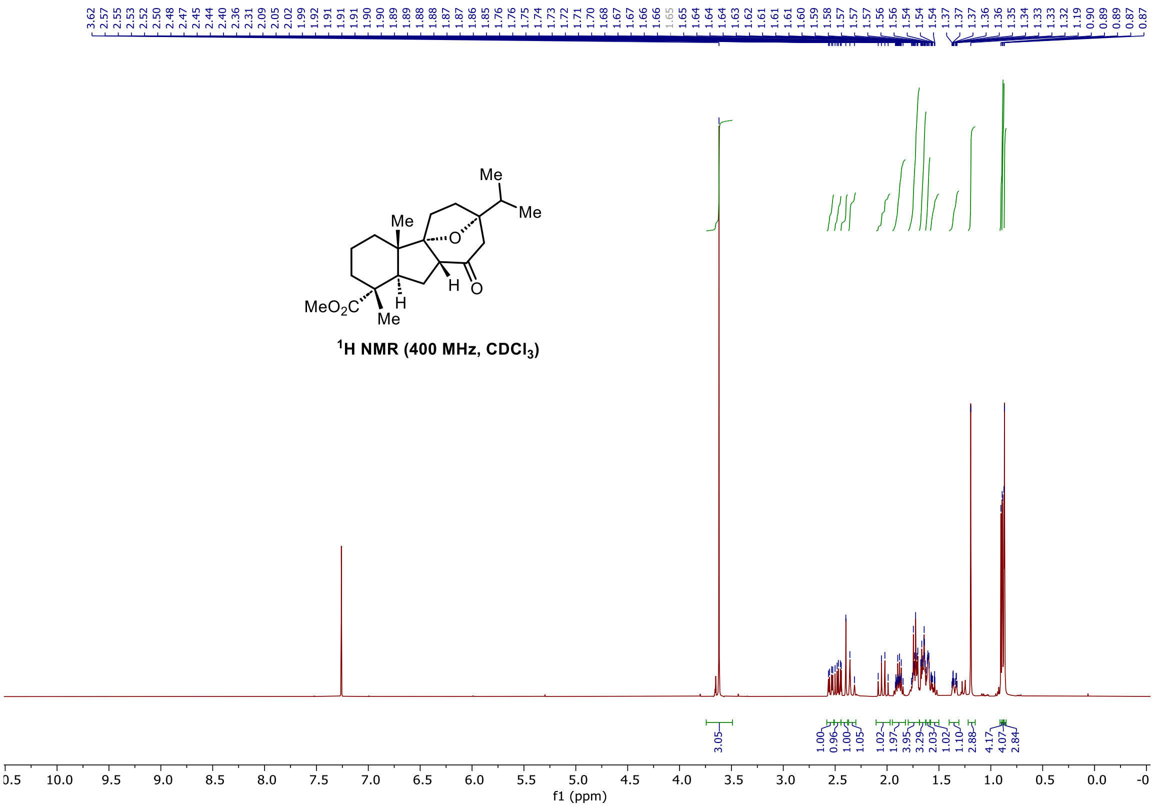
3.62 2.57 2.55 2.53 2.52 2.50 2.48 2.47 2.45 2.44 2.40 2.36 2.31 2.09 2.05 2.02 1.99 1.92 1.91 1.91 1.90 1.90 1.89 1.89 1.88 1.87 1.87 1.86 1.85 1.76 1.76 1.75 1.74 1.73 1.72 1.71 1.70 1.68 1.67 1.67 1.66 1.66 1.65 1.65 1.64 1.64 1.63 1.62 1.61 1.61 1.60 1.59 1.58 1.57 1.57 1.57 1.56 1.56 1.54 1.54 1.54 1.37 1.37 1.37 1.36 1.36 1.35 1.34 1.33 1.33 1.32 1.19 0.90 0.89 0.87 0.87
Me
Me
Me
O
MeO2C
H
H
O
Me
^{1}H NMR (400 MHz, CDCl$_3$)
3.05
1.00 0.96 1.00 1.05 1.02 1.97 3.95 3.29 2.03 1.02 1.10 2.88 4.17 4.07 2.84
f1 (ppm)

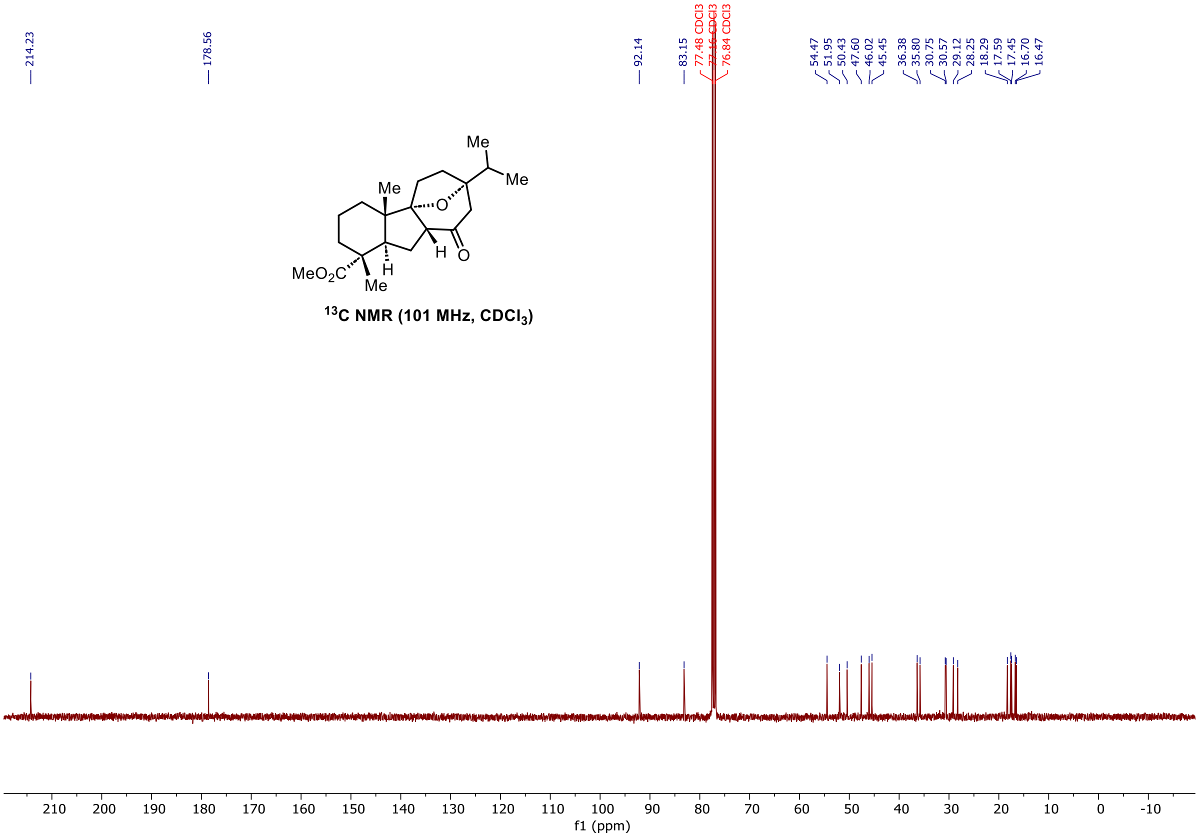
214.23
178.56
92.14
83.15
77.48 CDCl3
77.16 CDCl3
76.84 CDCl3
54.47 51.95 50.43 47.60 46.02 45.45 36.38 35.80 30.75 30.57 29.12 28.25 18.29 17.59 17.45 16.70 16.47
Me
Me
Me
O
MeO2C
H
H
O
Me
^{13}C NMR (101 MHz, CDCl$_3$)
f1 (ppm)
